# Manganese nutrient mitigates ammonia, arsenic toxicity and high temperature stress using gene regulation via NFkB mechanism in fish

**DOI:** 10.1038/s41598-024-51740-1

**Published:** 2024-01-13

**Authors:** Neeraj Kumar, Supriya Tukaram Thorat, Sanjivkumar Angadrao Kochewad, Kotha Sammi Reddy

**Affiliations:** ICAR-National Institute of Abiotic Stress Management, Malegaon, Baramati, Pune, 413115 India

**Keywords:** Zoology, Animal physiology, Ichthyology

## Abstract

The ongoing challenges of climate change and pollution are major factors disturbing ecosystems, including aquatic systems. They also have an impact on gene regulation and biochemical changes in aquatic animals, including fish. Understanding the mechanisms of gene regulation and biochemical changes due to climate change and pollution in aquatic animals is a challenging task. However, with this backdrop, the present investigation was conducted to explore the effects of arsenic (As) and ammonia (NH_3_) toxicity and high-temperature (T) stress on gene regulation and biochemical profiles, mitigated by dietary manganese (Mn) in *Pangasianodon hypophthalmus*. The fish were exposed to different combinations of As, NH_3_, and T, and fed with dietary Mn at 4, 8, and 12 mg kg^−1^ to evaluate the gene expression of immunity, antioxidative status, cytokine, and NfKB signaling pathway genes. HSP 70, cytochrome P450 (CYP 450), metallothionein (MT), DNA damage-inducible protein (DDIP), caspase (CAS), tumor necrosis factor (TNFα), toll-like receptor (TLR), interleukin (IL), inducible nitric oxide synthase (iNOS), catalase (CAT), superoxide dismutase (SOD), and glutathione peroxidase (GPx) were noticeably highly upregulated by As + NH_3_ + T stress, whereas Mn diet at 8 mg kg^−1^ downregulated these genes. Further, total immunoglobulin (Ig), myostatin (MYST), somatostatin (SMT), growth hormone (GH), growth hormone regulator 1 and β, insulin-like growth factors (IGF1X1 and IGF1X2) were significantly upregulated by Mn diets. The biochemical profiles were highly affected by stressors (As + NH_3_ + T). The bioaccumulation of arsenic in different tissues was also notably reduced by Mn diets. Furthermore, the infectivity of the fish was reduced, and survival against pathogenic bacteria was enhanced by Mn diet at 8 mg kg^−1^. The results of the present investigation revealed that dietary Mn at 8 mg kg^−1^ controls gene regulation against multiple stressors (As, NH_3_, As + NH_3_, NH_3_ + T, As + NH_3_ + T) in fish.

## Introduction

Abiotic factors play a pivotal role in the decline of agricultural and allied sector production. Climate change, pollution, and degraded water quality stand out as major abiotic influencers shaping the life patterns of aquatic organisms, including fish^1,2^. Ammonia holds a crucial position in the nitrogen cycle, undergoing conversion into nitrite (NO_2_) by Nitrosospira and Nitrosomonas bacteria in aquatic systems through the nitrification process. Additionally, it originates from fish waste, high-protein diets, and metabolic processes, contributing to the presence of ammonia in aquatic systems^3,4^. The breakdown of amino acids, pyrimidines, and purines also generates ammonia^5^, existing in two forms: unionized ammonia (NH_3_) and ionized ammonium (NH_4_^+^)^6^. Ammonia toxicity can lead to noticeable reductions in growth performance^2^, immunity, tissue erosion, neurotoxicity, oxidative stress, and ultimately result in high mortality^7^.

Naturally occurring arsenic, typically harmless in its natural state, can undergo transformation into inorganic arsenic, thereby contaminating groundwater sources used for drinking or irrigating crops. The accumulation of arsenic from one trophic level to another level, depends not only on the total arsenic content but also significantly on its bioavailability^8^. The chemical forms of arsenic, such as inorganic and organic forms present in crops, vegetables, and fish, play a crucial role in determining bioavailability, which is essential for estimating its toxicity. Humans can also uptake arsenic from contaminated sources such as rice, vegetables, milk, and meat. Consequently, 'plant–human' and 'plant–animal–human' represent potential food chain pathways for arsenic accumulation^9,10^.

It is also considered to be consumption of even low dose of arsenic can cause deadly diseases, including cancer^11,12^. In all around the world, such as in India, Bangladesh, Argentina, China, Ghana, USA, and Vietnam, more than 200 million peoples are at high risk^13,14^. Further, in Bangladesh, 43,000 peoples die annually due to arsenic pollution^14^.

Fish are classified as poikilothermic animals; however, even with slight temperature variations, their physiology undergoes abrupt changes. These changes include alterations in growth, metabolism, food consumption, thermal tolerance, and an inability to maintain internal homeostasis in response to the fluctuating external environment^15,16^. Moreover, elevated temperatures diminish the availability of oxygen to aquatic animals, creating challenges in meeting metabolic demands, especially as the water flow rate increases across the gills^1^.

Interestingly, manganese (Mn) plays a vital role as an essential micronutrient in the growth and development of the vertebral column, serving as an antioxidant and acting as a cofactor for numerous enzymes^17^. Typically, the requirement for Mn is met through waterborne sources, but additional supplementation is necessary to fulfil the physiological needs of the fish^18,19^. Therefore, Mn supplements are provided to meet the physiological requirements and metabolic scope of the fish. A deficiency in Mn for fish can lead to retarded growth performance, skeletal deformities (dwarfism), eye lens cataracts, decreased activities of copper-zinc superoxide dismutase (Cu–Zn-SOD), manganese superoxide dismutase complex (Mn-SOD), and reduced reproductive performance^19–21^. Mn is primarily located in the mitochondria and plays a crucial role in activating several enzymes, including decarboxylases, kinases, hydrolases, and transferases. Key manganese metallo-enzymes, such as pyruvate carboxylase, catalyze the conversion of pyruvate to oxaloacetate^22^.

Apoptosis is a programmed cell death crucial for regular cell repair, cellular function, immune and hormone-related gene development, and chemical cell death in all organisms, including fish^23^. Cytokines, serving as essential signaling molecules, are released during both physiological and pathological conditions. They play a role in stress responses and modulate the host's inflammatory response and immunobiological mechanisms^24,25^. Furthermore, NF-kB regulates and controls the transcription of genes related to immune cells, inflammation, proliferation, the cell cycle, and cell death^26^.

*Pangasianodon hypophthalmus* exhibits great potential as a fish species suitable for cultivation in challenging conditions, displaying tolerance to high abiotic and biotic stress^16,27,28^. Moreover, it is an ideal species for studying gene regulation involved in both abiotic and biotic stress^2^. Consequently, the present investigation aims to study the role of manganese in mitigating arsenic and ammonia toxicity, as well as high-temperature stress. This study also explores gene regulation associated with abiotic and biotic stress in response to dietary manganese in *P. hypophthalmus*.

## Materials and methods

### Ethics statement

The Research Advisory Committee (RAC) of the Institute (ICAR-National Institute of Abiotic Stress Management, Baramati, Pune) has approved the experimental procedures, and this study adheres to the Animal Research: Reporting of In Vivo Experiments (ARRIVE) guidelines. All methods were conducted in strict accordance with the relevant guidelines and regulations.

### Experimental animal and design

*Pangasianodon hypophthalmus* specimens with an average weight of 6.71 ± 0.52 g and a length of 5.12 ± 0.17 cm was utilized in the current investigation. These fish were sourced from the NIASM farm pond and were in a healthy condition. At Prior to the commencement of the experiment, a two-week acclimatization period was provided to the fish in a Fiberglass Reinforced Plastic (FRP) tank. During this acclimatization period, the fish received regular feeding and other necessary maintenance. Subsequently, the eighteen fish were evenly distributed in a plastic rectangular tank with a capacity of 150 L. The experiment was designed with 12 treatments, each replicated in triplicate, employing a Completely Randomized Design (CRD). The treatments followed as 1. Control 2. As exposed group 3. Ammonia exposed group 4. Concurrent exposure to arsenic and ammonia group 5. Concurrent exposure to ammonia and high temperature group 6. Concurrent exposure to arsenic, ammonia and high temperature group 7. Group fed with Mn at 4 mg kg^−1^ diet 8. Group fed with Mn at 8 mg kg^−1^ diet 9. Group fed with Mn at 12 mg kg^−1^ diet 10. Group fed with Mn at 4 mg kg^−1^ diet and concurrently exposed to arsenic, ammonia and high temperature 11. Group fed with Mn at 8 mg kg^−1^ diet and concurrently exposed to arsenic, ammonia and high temperature 12. Group fed with Mn at 12 mg kg^−1^ diet and concurrently exposed to arsenic, ammonia and high temperature. The details of the treatment is shown in Table 1. The experimental diets were administered twice daily to the fish at 9:00 AM and 5:00 PM. Continuous aeration was maintained throughout the experiment using an aerator. Daily removal of uneaten feed and faecal matter was carried out through siphoning. Periodic analysis of water quality parameters was conducted using the APHA method^29^, and the results consistently fell within acceptable ranges throughout the experiment. Every alternate day, 2/3rd of the water in the tank was manually replaced. Additionally, (NH_4_)_2_SO_4_ was added as a source of ammonia toxicity (NH_3_), and sodium arsenite, NaAsO_2_, was introduced as a source of arsenic. The concentrations used were Ammonium sulfate (1/10th of LC_50_, 2.0 mg L^−1^ of (NH_4_)_2_SO_4_)^2^ and As (1/10th of LC_50_, 2.68 mg L^−1^ of arsenic)^1^. The water temperature was maintained at a high level (34 °C) throughout the experiment to induce stress. Four iso-caloric (365 kcal/100 g) and iso-nitrogenous (35% crude protein) pelleted diets containing manganese were prepared. The feed ingredients included wheat flour, groundnut meal, soybean meal, and fish meal. Cod liver oil, lecithin, vitamin C, and other labile nutrients were added after heating the feed ingredients. A manganese-free mineral mixture was manually prepared. Proximate analysis was conducted using the AOAC method^30^, while ether extract (EE) was determined through solvent extraction, crude protein by nitrogen content, and ash content by using a muffle furnace at 550 °C. Total carbohydrate content was calculated using the formula 100—(CP% + EE% + Ash %+moisture). Gross energy was determined using the Halver method^31^ (Table 2).

**Table 1:** experimental design of present investigation.

S. No	Details of the treatments	Notation
1	Control	Ctr
2	Fed with control diet and exposure to arsenic	As
3	Fed with control diet and exposure to ammonia	NH_3_
4	Fed with control diet and concurrently exposure to arsenic and ammonia	As + NH_3_
5	Fed with control diet and concurrent exposure to ammonia and high temperature	NH_3_ + T
6	Fed with control diet and concurrent exposure to arsenic, ammonia and high temperature	As + NH_3_ + T
7	Fed with manganese at 4 mg kg^−1^ diet	Mn at 4 mg kg^−1^ diet
8	Fed with manganese at 8 mg kg^−1^ diet	Mn at 8 mg kg^−1^ diet
9	Fed with manganese at 12 mg kg^−1^ diet	Mn at 12 mg kg^−1^ diet
10	Fed with manganese at 4 mg kg^−1^ diet and concurrent exposure to arsenic, ammonia and high temperature	Mn at 4 mg kg^−1^ diet + As + NH_3_ + T
11	Fed with manganese at 8 mg kg^−1^ diet and concurrent exposure to arsenic, ammonia and high temperature	Mn at 8 mg kg^−1^ diet + As + NH_3_ + T
12	Fed with manganese at 12 mg kg^−1^ diet and concurrent exposure to arsenic, ammonia and high temperature and	Mn at 12 mg kg^−1^ diet + As + NH_3_ + T

**Table 2:** Ingredients composition and proximate analysis of experimental diets (% dry matter) of manganese (Mn), fed to *Pangasianodon hypophthalmus* for 105 days.

Feed ingredients	Mn-0 mg kg^−1^ diet	Mn-4 mg kg^−1^ diet	Mn-8 mg kg^−1^ diet	Mn-12 mg kg^−1^ diet
Soybean meal^a^	35.5	35.5	35.5	35.5
Fish meal^a^	25	25	25	25
Groundnut meal^a^	10	10	10	10
Wheat flour^a^	17.2	17.196	17.192	17.188
Sunflower oil^a^	4.5	4.5	4.5	4.5
Cod liver oil^a^	1.5	1.5	1.5	1.5
CMC^b^	2	2	2	2
Vitamin and mineral mix*	2	2	2	2
Vitamin C^c^	0.3	0.3	0.3	0.3
Lecithin^b^	2	2	2	2
Mn	0	0.004	0.008	0.012
Proximate composition of the diets				
Crude protein (CP)	35.34 ± 0.39	35.16 ± 0.08	35.43 ± 0.18	35.17 ± 0.02
Ether extract (EE)	8.23 ± 0.09	8.57 ± 0.22	8.72 ± 0.31	8.34 ± 0.10
Total carbohydrate (TC)	40.37 ± 0.36	40.89 ± 0.68	40.58 ± 0.41	40.13 ± 0.08
Organic matter (OM)	92.05 ± 0.08	92.26 ± 0.28	91.98 ± 0.07	91.81 ± 0.02
Dry matter (DM)	91.90 ± 0.13	92.36 ± 0.30	92.74 ± 0.04	91.83 ± 0.13
Digestible energy (DE)	363.47 ± 0.93	364.20 ± 1.95	365.03 ± 0.99	364.59 ± 0.85

^a^Procured from local market, ^b^Himedia Ltd, Himedia Ltd, ^c^SD Fine Chemicals Ltd., India.

*Manual prepared Vitamin mineral mixture; Composition of vitamin mineral mix (quantity/250 g starch powder): vitamin A 55,00,00 IU; vitamin D3 11,00,00 IU; vitamin B1:20 mg; vitamin E 75 mg; vitamin K 1,00 mg; vitamin B12 0.6 mcg; calcium pantothenate 2,50 mg; nicotinamide 1000 mg; pyridoxine: 100 mg; Zn 500 mg; I 1,00 mg; Fe 750 mg; Cu 200 mg; Co 45 mg; Ca 50 g; P 30 g; Se: 2 ppm.

Digestible energy (DE) (Kcal/100 g)=(%CP×)+(%EE×9)+(TC×4)

Data expressed as mean ± SE, *n* = 3.

### Tissue homogenate preparation and blood collection

The gill, muscle, brain, liver, and kidney were dissected from anesthetized fish (clove oil, 50 µl L^−1^) under aseptic conditions. The chilled sucrose (5% w/v, 0.25 M) and EDTA solution (1 mM) were used as homogenates for tissue homogenization using a homogenizer (Omni Tissue Master Homogenize, Kennesaw, GA) for enzyme analysis. The gene expression and quantification, the liver and muscle tissues samples were processed with liquid nitrogen. For the enzymes analysis, the tissues were homogenized and centrifuged at 5,000 × g for 15 min at 4 °C to homogenated samples. The supernatants were collected and stored at -20 °C until further analysis. During dissection, the blood (3 fish) was also collected from the same fish of each tank and serum (5 fish) was processed from the same collected blood. Lowry protein assay^32^ was used for tissue protein analysis.

### RNA isolation and quantification and cDNA synthesis and quantitative PCR

The TRIzol method was employed for total RNA isolation from the liver tissue of *P. hypophthalmus*. Liquid nitrogen was utilized for the homogenization of the liver tissue. Subsequently, chloroform was added to the homogenized samples, and the mixture was incubated for 5 min to allow for phase separation. The resulting solution was then centrifuged to separate the RNA, followed by the addition of 75% ethanol and air drying. The RNA pellet was dissolved in free water and stored at -80 °C for future use. To assess RNA integrity, 1% agarose gel electrophoresis was conducted, and the RNA bands were visualized using a Gel documentation system (ChemiDocTM MP imaging system, Bio-Rad). RNA quantification was performed using a NanoDrop spectrophotometer (Thermo Scientific). Revert Aid First Strand cDNA synthesis kit (Thermo Scientific) was utilized. DNase I was employed to remove trace amounts of DNA. The reaction mixture, consisting of oligo dT primers (15 pmol) and RNA template (100 ng) in 12 µl, was heated for 5 min at 65 °C and then chilled on ice. Subsequently, 1.0 µl of reverse transcriptase enzyme, 2 µl dNTP Mix (10 mM), and 1 µl Ribo Lock RNase Inhibitor (20 U/µL) were added to the chilled mixture, followed by a brief centrifugation. The reaction mixture was incubated for 42 min at 60 °C, then at 70 °C for 5 min, and the synthesized cDNA was stored at -20 °C. β-actin was used as a reference for confirming the synthesized cDNA. Real-time PCR was conducted using SYBR green and gene-specific primers (Bio-Rad). The quantification protocol included an initial denaturation for 10 min at 95 °C, followed by 39 cycles of cDNA amplification, denaturation at 95 °C for 15 s, and annealing at 60 °C for 1 minute^33^. Details of the primers are recorded in Table 3.

**Table 3:** Details of primer for relative quantitative real-time PCR.

Gene	Primer sequence (5′–3′)	Accession number
SOD	F-GTCCATCTTACCCGGTGCCC R-CGAGAGAAGACCCGGAACGC	XM_034299545.1
CAT	F-AGCAGGCGGAGAAGTACCCA R-GCTGCTCCACCTCAGCGAAA	XM_026919141.2
GPx	F- GTCACTGCAGGATGCAACAC R- TTGGAATTCCGCTCATTGAT	XM_026947312.2
HSP 70	F- CTCCTCCTAAACCCCGAGTC R- CCACCAGCACGTTAAACACA	XM_026934573.2
iNOS	F-ACACCACGGAGTGTGTTCGT R-GGATGCATGGGACGTTGCTG	XM_026931613.2
DNA damage inducible protein	F-CACCTTCGCCCTCGAAGTCT R-GCTCGGGTGAGGTCTCTCAG	XM_026938137.2
TNFα	F-TGGAGTTCTGCTTGCCGTGG R-GCAGCCTTTGCAGTCTCGGA	XM_026942329.2
TLR	F: TCACCACGAACGAGACTTCATCC R : GACAGCACGAAGACACAGCATC	XM_026916808.2
Ghr1	FTATTGGCTACAGCTCGCCGC R-AATCACCCCGACTGTGCTGC	XM_034306157.1
Ghrb	F-TTGAGCTTTGGGACTCGGAC R-CGTCGATCTTCTCGGTGAGG	XM_026942987.2
IGF-1X1	F-GCAACGGCACACAGACACGC R-CAGACGTTCCCTCACCATCCTCT	XM_034313382.2
IGF-1X2	F-CGAGAGCAACGGCACACAGA R-TTCTGATGGACCTCCTTACAAGATG	XM_034313383.2
IL	F-AGCAGGATCCATCAAAGTGG R-GTGCTCCAGCTCTCTGGGTA	XM_026918084.2
Ig	F-GGCCAGTAATCGTACCTCCA R-CTTCGTAAGGTCCCCACTGA	XM_026923540.2
MYST	F-GGGAAAGACCTGGCCGTGAC R-TCGAGGCCGGATTCTCGTCT	XM_026910492.2
SMT	F-CTCTGGGTGGCAGAATGAAT R-AACATGAAGAGAACGTTTTCCAG	XM_026921272.2
GH	F-CCCAGCAAGAACCTCGGCAA R-GCGGAGCCAGAGAGTCGTTC	GQ859589.1
Cas3b	F-AGCTTTCCGTGAGCTGGGCT R-TGGCTGACTTGCTGTGGTCCT	NC_047601.1
Na^+^K^+^ATPase	F-AACTACAAGCCCACGTACCA R-CTTGCCAGCCTTAAAGCCAA	XM_026923907.3
β-Actin	F-CAGCAAGCAGGAGTACGATG R-TGTGTGGTGTGTGGTTGTTTTG	XM_031749543.1

SOD: Superoxide dismutase; CAT: Catalase; GPx: Glutathione peroxidase; HSP: Heat shock protein; iNOS: Nitric oxide synthase; TNFα: Tumor necrosis factor; TLR: Toll like receptor; Ghr: Growth hormone receptor; IL; Interleukin; Ig: Immunoglobulin; MYST: myostatin SMT; Somatostatin; CYP P450: Cytochrome P450; MT: Metallothionine; Cas 3a and 3b: caspase 3; GH: Growth hormone; IGF1 and 2: Insulin like growth factor.

### Genes

The genes were investigated in liver tissues in this study viz. catalase (CAT), glutathione-s-transferase (GST), superoxide dismutase (SOD), nitric oxide synthase (iNOS), heat shock protein (HSP 70), Caspase 3a (CAS 3a and 3b), cytochrome P450 (CYP 450), tumor necrosis factor (TNFα), toll like receptor (TLR), metallothionine (MT), growth hormone receptor (Ghr1 and Ghrb), interleukin (IL), immunoglobulin (Ig), insulin like growth factor 1 and 2 (IGF1X1 and IGF1X2), somatostatin (SMT), myostatin (MYST), and growth hormone (GH), studied for real-time quantification.

### Antioxidant enzyme activities

Superoxide dismutase (SOD) (EC 1.15.1.1) activities in different fish tissues were determined by Misra and Fridovich^34^. Catalase (EC 1.11.1.6) was determined as followed as a procedure of Takahara et al^35^. The glutathione S-transferase (GST) (EC 2.5.1.18) was determined as per the procedure of Habing et al^36^. Glutathione peroxidase (GPx) (EC 1.11.1.9) activity was accomplished following the method of Paglia and Valentine^37^.

### Neurotransmitter enzyme activities

Hestrin modified by Augustinsson^38^ method was applied to determine the acetylcholine esterase activities (AChE) (EC. 3.1.1.7) in brain tissue.

### Lipid peroxidation (LPO) and Vitamin C

Uchiyama and Mihara^39^ method was followed to determine the LPO in liver and kidney tissues. Similarly, Roe and Keuther^40^ used to determine the Vitamin C in brain and muscle tissues.

#### Hematological parameters

Blood was drawn from the caudal peduncle region of the fish using heparinised syringe. Indices measured included erythrocyte count (RBC), hemoglobin concentration (Hb), WBC (total leucocyte count) and the procedures were based on unified methods for hematological examination of fish.

#### Immunological attributes

Total serum protein, albumin, globulin, and A:G ratio was determined using the protein estimation kit. Secombes^41^, with some modification by Stasiack and Baumann^42^ used for the estimation of respiratory burst activity. The blood glucose was determined using Nelson^43^ and Somoyogi^44^. Moreover, Quade and Roth^45^, with some modifications^46^ and Anderson and Siwicki^47^ were applied for the determination of myeloperoxidase and total immunoglobulin.

#### Cortisol

Serum cortisol was determined using ELISA kit (Commercially available Cortisol EIA kit, catalogue no. 500360, Cayman Chemicals, USA). The assay was performed as per instruction provided with the kit using ELISA plate reader (Biotek India Pvt. Ltd.).

#### Arsenic and manganese analysis from fish tissues and experimental water

Liver, muscle, gill, brain, and kidney were collected to determine in arsenic concentration. Whereas, Mn concentration was determined in the feed and fish muscle. The tissues and diets were processed in a microwave digestion system (Microwave Reaction System, Multiwave PRO, Anton Paar GmbH, Austria, Europe) using Inductively Coupled Plasma Mass Spectrometry (ICP-MS) (Agilent 7700 series, Agilent Technologies, USA) as followed the method of Kumar et al.^48,49^.

#### Alkaline single-cell gel electrophoresis (SCGE)/Comet assay

Alkaline single cell gel electrophoresis/comet assay was applied for determination of DNA damage in kidney tissue using Ali et al^50^. with slight modification^51^. The slides coating and other procedure were followed the above method. Then prepared slides for genotoxicity were analysed in fluorescent microscope (Leica Microsystems Ltd, DM 2000, Heerbrugg, Switzerland). The position of DNA damage was captured using the microscope and analyzed using Open comet. The parameter selected for quantification of DNA damage was percent tail DNA (i.e., % tail DNA = 100% head DNA) as determined by the software.

#### Aspartate aminotransferase (AST) and alanine aminotransferase (ALT), Lactate dehydrogenase (LDH), and malate dehydrogenase (MDH)

AST (E.C.2.6.1.1) and ALT (E.C.2.6.1.2) were determined using Wooten^52^ method. Similarly, LDH activities were determined using Wroblewski and Ladue^53^. Similarly, MDH was determined using Ochoa^54^. A similar reaction mixture was used except for substrate oxaloacetate instead of sodium pyruvate.

#### Growth performance

The growth performance was determined by evaluating the following method. The sampling/weighing of the fish was observed by every 15 days up to 105 days.

$${\text{FCR}}\; = \;{\text{Total}}\;{\text{dry}}\;{\text{feed}}\;{\text{intake}}\;\left( g \right)/{\text{Wet}}\;{\text{weight}}\;{\text{gain}}\;\left( g \right)$$

$${\text{SGR }} = { 1}00 \, \left( {{\text{ln FBW}} - {\text{ln IBW}}} \right)/{\text{ number of days}}$$

$${\text{Weight gain }}\left( \% \right) \, = {\text{ Final body weight }}\left( {{\text{FBW}}} \right) - {\text{Initial body weight }}\left( {{\text{IBW}}} \right)/{\text{Initial body weight }}\left( {{\text{IBW}}} \right) \, \times {1}00$$

$${\text{Relative feed intake}}, \, \left( {{\text{FI}}} \right) \, \left( {\% /{\text{d}}} \right) \, = { 1}00 \, \times \, \left( {{\text{TFI}}/{\rm I}{\text{BW}}} \right)$$

$${\text{PER}} = {\text{ Total wet weight gain }}\left( {\text{g}} \right)/{\text{crude protein intake }}\left( {\text{g}} \right)$$

$${\text{Thermal growth coefficient}}, \, \left( {{\text{TGC}}} \right) \, = \, \left( {{\text{FBW}}^{{{1}/{3}}} {-}{\text{ IBW}}^{{{1}/{3}}} } \right) \, \times \, \left( {\Sigma {\text{D}}0} \right)^{{ - {1}}} ,{\text{ where }}\Sigma {\text{D}}0{\text{ is the thermal sum }}\left( {{\text{feeding days }} \times {\text{ average temperature}}, \, ^{ \circ } {\text{C}}} \right)$$

$${\text{Daily growth index}},{\text{ DGI }}\left( \% \right) \, = \, \left( {{\text{FBW}}^{{{1}/{3}}} {-}{\text{ IBW}}^{{{1}/{3}}} } \right)/{\text{days }} \times { 1}00$$

#### Challenge study with *Aeromonas hydrophila*

After 105 days of the feeding trial, 8 fishes per replicates in each treatment were challenged with virulent *A. hydrophilla* (Lot no. 637–51-5 and Ref 0637P, HiMedia, Mumbai). *A. hydrophilla* was grown on a nutrient broth for 24 h at 37 °C in a BOD incubator and harvested by centrifuging the culture broth at 10,000 × g for 10 min at 4 °C. The cells were then washed thrice in sterile PBS (pH 7.2), and the final concentration was maintained at 10^8^ CFU ml^−1^. The fish were intraperitoneally injected with 0.15 ml of bacterial suspension in each treatment group. The fish mortality in each treatment group was recorded up to 7 days of challenge study. The tissues were dissected out from morbid fish for confirmation of *A. hydrophilla* as a causative agent for death.

$${\text{Cumulative mortality }}\left( \% \right) \, =\frac{{\text{ Total mortality in each treatment after challenge }}}{{\text{ Total no}}.{\text{ of fish challenged for the same treatments}}} \times {1}00$$

$${\text{Relative }}\% {\text{ survival }} = \frac{{\text{ Mortality }}\left( \% \right){\text{ Control }}{-}{\text{ Mortality }}\left( \% \right){\text{ Treatment }}}{{\text{ Mortality }}\left( \% \right){\text{ Control}}}\times { 1}00$$

#### Statistics

The data were analysed using Statistical Package for the Social Sciences (SPSS) version 16 software. The data were tested for normality and homogeneity of variance using Shapiro–Wilk’s and Levene's test and Shapiro–Wilk's test, respectively. One way ANOVA (analysis of variance) using Duncan’s multiple range tests were applied in the present study. The data were analysed and significant at *p* < 0.05.

### Ethics approval

The Institute (ICAR-National Institute of Abiotic Stress Management, Baramati, Pune) Research Advisory Committee (RAC) has approved the experimental procedures and this study compliance with Animal Research: Reporting of In Vivo Experiments (ARRIVE) guidelines.

### Consent to participate

All authors are aware and agree with this submission for publication.

## Results

3.1. Effect of Mn on cortisol levels.

**Figure 1:**
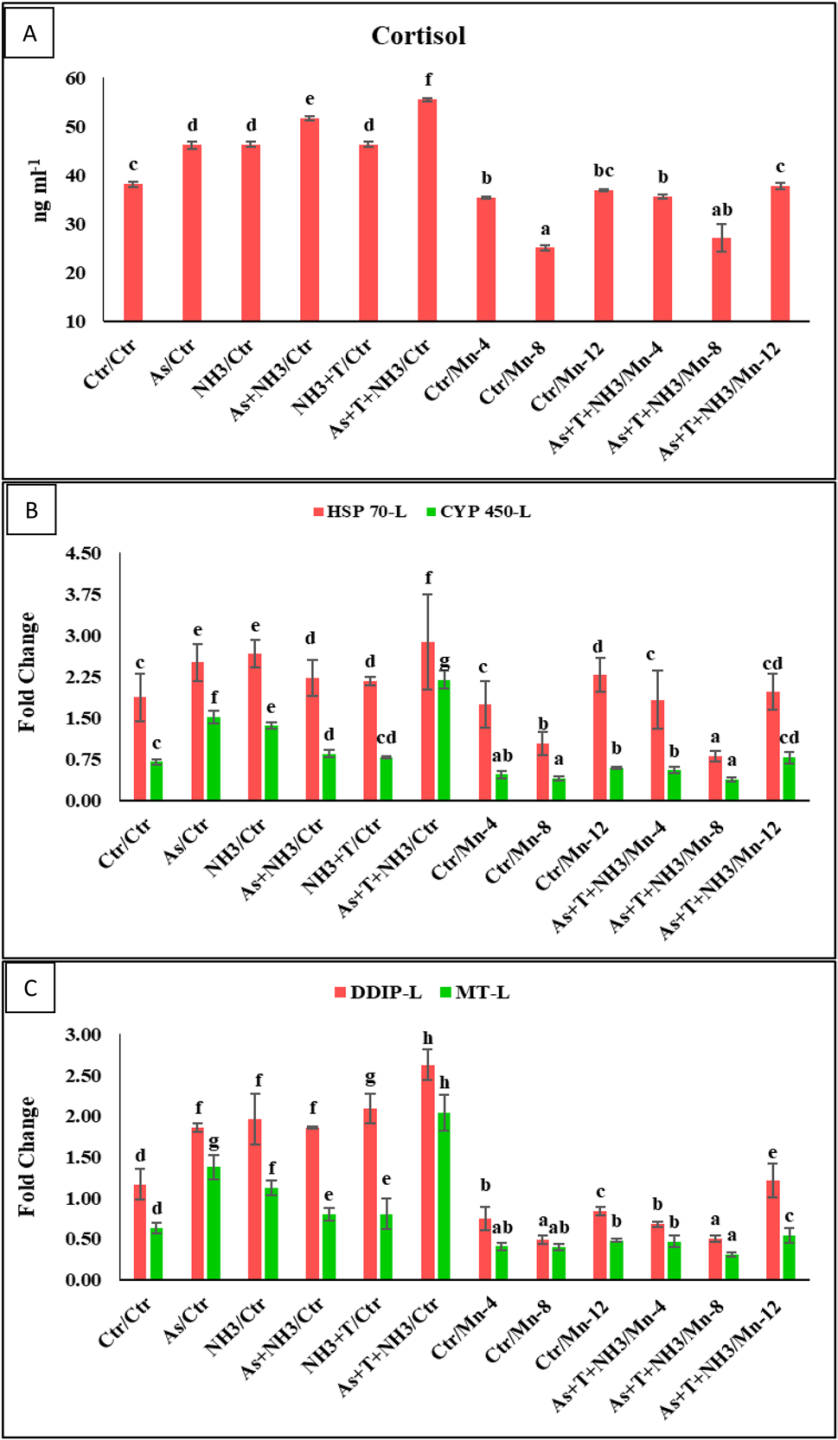
Manganese diets control the cortisol and gene expression of HSP 70, CYP 450, DNA damage inducible protein (DDIP) and metallothionine (MT) against multiple stressors in fish. Within endpoints and groups, bars with different superscripts differ significantly (a–h) Cortisol (*p* = 0.0025), HSP-L (*p* = 0.0017), CYP 450 *p* = 0.0013), DDIP *p* = 0.0011), MT *p* = 0.0002). Data expressed as Mean ± SE (*n* = 3).

In the present investigation, cortisol levels were assessed in response to dietary manganese (Mn) at 4, 8, and 12 mg kg^−1^ diet fed to *P. hypophthalmus*. The fish were reared under normal conditions as well as under the arsenic and ammonia pollution, coupled with high-temperature stress, over a period of 105 days. The corresponding data are illustrated in Fig. 1A. Cortisol levels exhibited a noticeable increase (*p* = 0.0025) in the group subjected to concurrent exposure to arsenic, ammonia toxicity, and high-temperature stress, followed by the group exposed to arsenic and ammonia, when compared to the control and other groups. Furthermore, dietary manganese at 8 and 4 mg kg^−1^ diet, with or without stressors (As + NH_3_ + T), significantly reduced cortisol levels (*p* = 0.0025) compared to the control and other groups. However, manganese at 12 mg kg^−1^ diet did not exhibit an inhibitory effect on cortisol levels in fish reared under both control and stressor conditions.

### Effect of Mn on Heat shock protein (HSP 70) and cytochrome P450 (CYP P450)

The expression of the HSP70 gene in liver tissue exhibited a significant increase (*p* = 0.0017) under concurrent exposure to ammonia and arsenic toxicity, along with high-temperature stress. This was followed by the group exposed to ammonia and high temperature, then arsenic and ammonia, ammonia alone, and arsenic alone groups, as compared to the control and Mn-supplemented groups. This observation held true for fish reared both in control conditions and under multiple stressors (As + NH_3_ + T). Interestingly, the group fed with a Mn-containing diet at 8 mg kg^−1^ with stressors, followed by the same group without stressors, and then the Mn at 4 mg kg^−1^ diet, exhibited a significant difference compared to the control and other groups (see Fig. 1B). Intriguingly, the expression of the CYP 450 gene in liver tissue was significantly upregulated (*p* = 0.0013) in response to a combination of different stressors (As + NH_3_ + T, NH_3_, As, As + NH_3_, and NH_3_ + T) compared to the control and Mn-supplemented groups (Mn at 4, 8, and 12 mg kg^−1^ in the diet). Conversely, the supplementation of dietary Mn at 8 and 4 mg kg^−1^, with or without stressors, resulted in a substantial downregulation of CYP 450 gene expression compared to the control and other groups (Fig. 1C).

### Effect of Mn on DNA damage-inducible protein (DDIP), DNA damage and metallothionine (MT)

DNA damage inducible protein (DDIP) exhibited a noticeable upregulation (*p* = 0.0011) with concurrent exposure to ammonia, arsenic, and high-temperature stress. This was followed by the group exposed to ammonia and high temperature, and then the arsenic and ammonia, ammonia alone, and arsenic alone groups, in comparison to the control and other groups. Surprisingly, dietary Mn at 8 mg kg^−1^ in the diet, with or without stressors, demonstrated the ability to substantially downregulated DDIP gene expression. This effect was followed by Mn at 4 mg kg^−1^ in the diet, as compared to the control and other groups. However, Mn at 12 mg kg^−1^ in the diet was not as effective in modulating DDIP gene expression against multiple stresses (Fig. 1C). Similarly, this study determined DNA damage in terms of tail DNA %, head DNA %, comet length, comet DNA, and head area, with the data recorded in Table 4. The tail DNA % was significantly highest in the group exposed to As + NH_3_ + T, NH_3_ + T, As + NH_3_, NH_3_, followed by the As alone group, compared to the control and Mn-supplemented groups. Further, the noticeably least tail DNA % was determined in the group fed with Mn at 4, 8, and 12 mg kg^−1^ in the diet without stressors, followed by the same feeding group but with stressors. Similarly, the results of head DNA % were inverse to tail DNA %. Interestingly, the expression of the metallothionein (MT) gene was substantially downregulated (*p* = 0.0002) with dietary Mn at 8 mg kg^−1^ in the diet, followed by Mn at 4 mg kg^−1^ in the diet, with or without stressors, in comparison to the control and other groups. However, concurrent exposure to As, NH_3_, and high temperature noticeably upregulated MT gene expression compared to arsenic and ammonia alone groups, in comparison to the control and Mn-supplemented groups (Fig. 1C).

**Table 4:** Effect of manganese (Mn) on DNA damage in *P. hypophthalmus* reared under control and multi stress condition.

Exposure/diets (mg kg^−1^)	Comet length	Comet DNA	Head area	Head DNA	Head DNA (%)	Tail DNA	Tail DNA (%)
Ctr/Ctr	35.0d ± 1.55	140,301 ± 23.98	942 ± 7.67	133,230 ± 26.67	94.96 g ± 1.98	7071 ± 21.87	5.04a ± 0.44
As/Ctr	30.0c ± 1.05	62,845 ± 15.34	156 ± 2.76	15,004 ± 27.09	23.87c ± 2.65	47,841 ± 20.71	76.13e ± 1.65
NH_3_/Ctr	28.0bc ± 0.67	46,116 ± 6.47	52 ± 1.45	4851 ± 11.45	10.52ab ± 1.22	41,265 ± 31.87	89.48f. ± 1.99
As + NH_3_/Ctr	24.0bc ± 0.93	48,760 ± 8.94	52 ± 1.21	5973 ± 16.32	12.25b ± 1.17	42,787 ± 16.69	87.75f. ± 2.67
NH_3_ + T/Ctr	48.0e ± 1.32	58,087 ± 9.27	51 ± 1.43	6723 ± 14.36	11.57b ± 1.05	51,364 ± 13.46	88.43f. ± 3.61
As + T + NH_3_/Ctr	23.0b ± 0.56	57,594 ± 11.54	52 ± 1.87	5573 ± 11.34	9.68a ± 0.56	52,021 ± 11.94	90.32f. ± 4.87
Ctr/Mn-4	9.93a ± 0.15	23,179 ± 6.34	40 ± 1.81	3886 ± 11.34	93.73 g ± 0.37	22,630 ± 22.76	6.27a ± 0.11
Ctr/Mn-8	22.0b ± 1.89	52,922 ± 7.93	377 ± 11.98	49,464 ± 25.87	93.47 g ± 1.85	3458 ± 11.33	6.53a ± 0.21
Ctr/Mn-12	30.0c ± 1.48	58,541 ± 17.45	798 ± 13.76	54,224 ± 16.74	92.63 g ± 1.25	4317 ± 17.56	7.37a ± 0.15
As + T + NH_3/_Mn-4	22.0b ± 1.01	48,292 ± 28.65	329 ± 11.71	40,212 ± 11.34	83.27e ± 1.67	8080 ± 9.32	16.73c ± 1.77
As + T + NH_3/_Mn-8	25.0bc ± 1.36	49,287 ± 27.65	380 ± 7.56	42,443 ± 11.87	86.11f. ± 1.15	6844 ± 13.23	13.89b ± 0.76
As + T + NH_3_/Mn-12	47.0 ± 1.76	71,698 ± 24.45	438 ± 8.36	41,374 ± 11.87	57.71d ± 1.09	30,324 ± 14.87	42.29d ± 0.65

### Effect of Mn on caspase 3a and 3b (Cas 3a and 3b)

**Figure 2:**
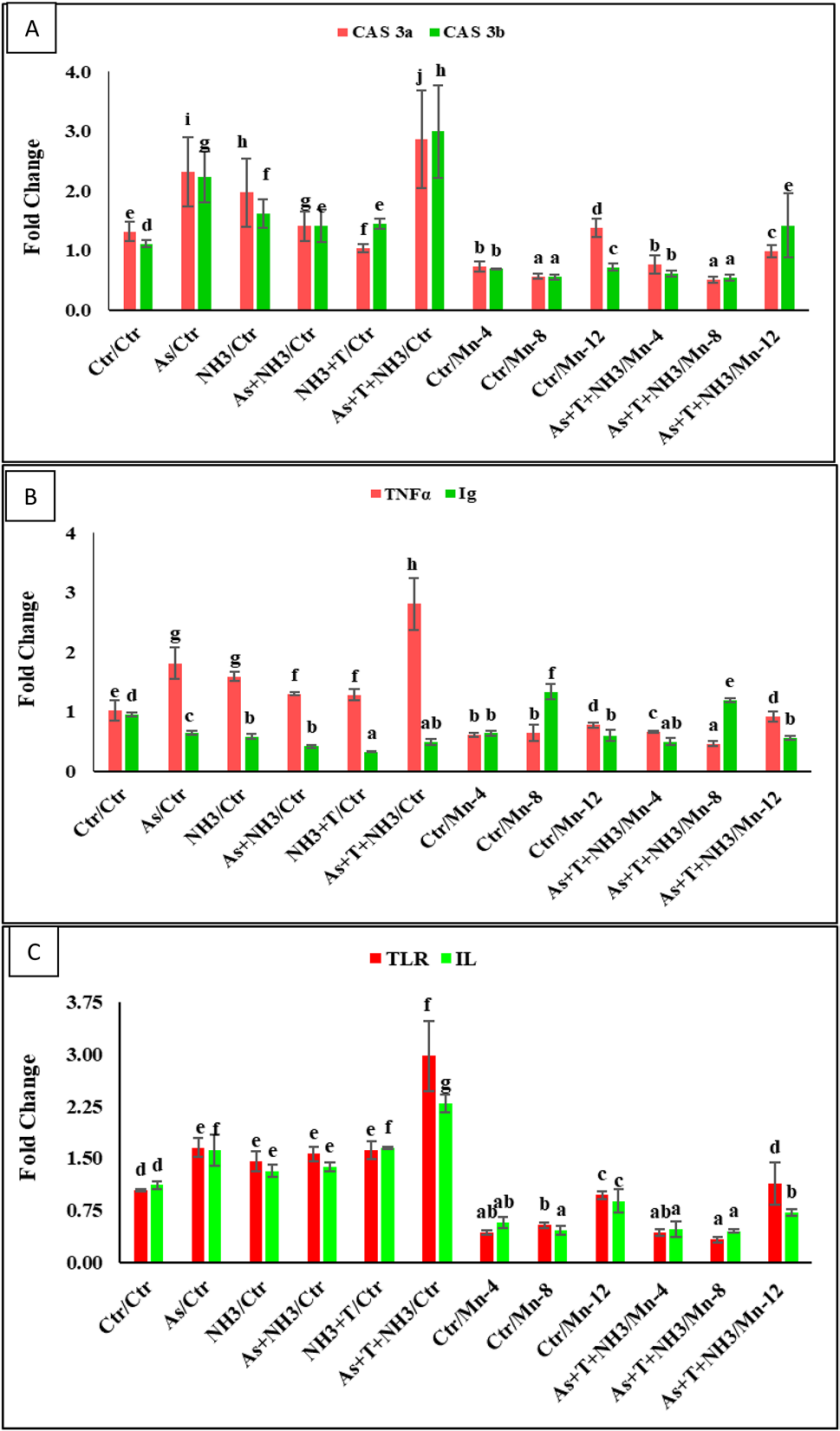
Manganese diets regulate the gene expression of Caspase 3a and 3b, TNFα, Ig, TLR and IL against multiple stressors in fish. Within endpoints and groups, bars with different superscripts differ significantly (a–g) Cas 3a *p* = 0.0052), Cas 3b *p* = 0.0003), TNFα *p* = 0.0023), Ig *p* = 0.0012), TLR *p* = 0.0046), IL *p* = 0.0029). Data expressed as Mean ± SE (*n* = 3).

The gene regulation of caspase 3a and 3b (Cas 3a and 3b) in liver tissue was significantly upregulated by concurrent exposure to ammonia, arsenic, and high-temperature stress. This upregulation was followed by the arsenic and ammonia alone group compared to the control and other treatments. Furthermore, the supplementation of Mn at 8 mg kg^−1^ diet, followed by Mn at 4 mg kg^−1^ diet, noticeably downregulated the Cas 3a (*p* = 0.0052) and 3b (*p* = 0.0003) gene regulations compared to the control and stressors group. However, Mn at 12 mg kg^−1^ in the diet was not effective for the gene regulation of Cas 3a and 3b (Fig. 2A).

### Effect of Mn on tumor necrosis factor (TNFα) and immunoglobulin (Ig), (TLR), and interleukin (IL) gene regulation

In the present investigation, the gene regulation of tumor necrosis factor (TNFα) and immunoglobulin (Ig) is presented in Fig. 2B. The gene regulation of TNFα was significantly downregulated (*p* = 0.0006) with the supplementation of dietary Mn at 8 mg kg^−1^ diet, with or without stressors. In contrast, TNFα was significantly upregulated, compared to the control and other groups, by concurrent exposure to As + NH_3_ + T, As, NH_3_, As + NH_3_, and As + T groups. Moreover, the Ig gene expression was noticeably upregulated (*p* = 0.0012) by the supplementation of Mn at 8 mg kg^−1^ diet, compared to other Mn-supplemented, control, and stressors groups. Further, the Ig gene expression was significantly downregulated with concurrent exposure to As + NH_3_ + T and NH_3_ + T, followed by As + NH_3_, NH_3_, and As alone groups, compared to the control and other groups. The toll-like receptors (TLR) (*p* = 0.0046) and interleukin (IL) (0.0029) were substantially upregulated by concurrent exposure to ammonia, arsenic, and high temperature, followed by NH_3_ + T, As + NH_3_, NH_3_, and As alone, in comparison to the control and Mn-supplemented groups. Furthermore, the gene regulation of TLR and IL was noticeably downregulated by Mn at 8 mg kg^−1^ diet, compared to the control and other groups (Fig. 2C).

3.6. Effect of Mn on catalase (CAT), superoxide dismutase (SOD), glutathione-s-transferase (GST) and glutathione peroxidase of biochemical activities and gene expressions. This is subtitle caption, Please make the subtitled like others.

**Figure 3:**
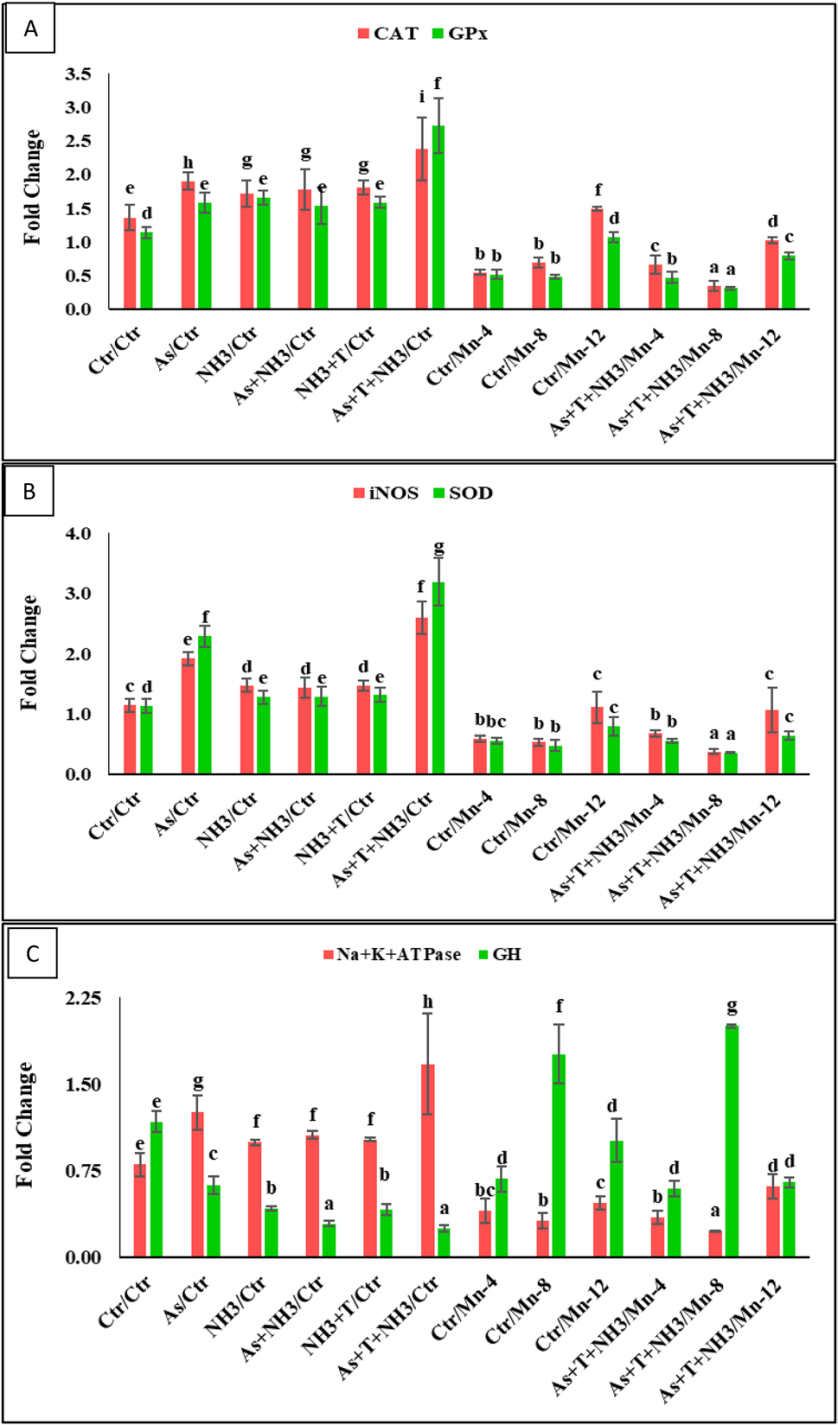
Manganese diets regulate the gene expression of Na^+^ K^+^ ATPase, GH, CAT, GPx, iNOS and SOD against multiple stress in fish. Within endpoints and groups, bars with different superscripts differ significantly (a–i) Na^+^ K^+^ ATPase *p* = 0.0022), GH *p* = 0.0016), CAT *p* = 0.001), GPx *p* = 0.014), iNOS *p* = 0.0036), SOD *p* = 0.0061). Data expressed as Mean ± SE (*n* = 3).

In the present investigation, the activities of anti-oxidative enzymes such as catalase (CAT), superoxide dismutase (SOD), glutathione-s-transferase (GST), and glutathione peroxidase were determined in the liver and gill tissues of *P. hypophthalmus* reared under arsenic and ammonia toxicity, along with high-temperature stress. The corresponding data are recorded in Table 5. CAT, GST, and GPx activities in the liver and gill were notably higher (*p* < 0.01) in the group concurrently exposed to As + NH_3_ + T, followed by NH_3_ + T, compared to the control and supplemented groups. Similarly, CAT, GPx, and GST activities in the liver and gill were also higher in the group exposed to arsenic and ammonia, compared to the control and Mn-supplemented groups (4 and 8 mg kg^−1^ diet). Interestingly, the supplementation of dietary Mn at 8 mg kg^−1^ diet, with or without stressors (As + NH_3_ + T), noticeably reduced CAT, GPx, and GST activities compared to the control and other treatment groups. The supplemented group with Mn at 4 mg kg^−1^ in the diet also effectively controlled CAT, GPx, and GST activities. Regarding SOD activities in the liver (*p* = 0.018) and gill (*p* = 0.037), they were significantly higher in the group treated with all stressors (As + NH_3_ + T, NH_3_ + T, As + NH_3_, NH_3_, and As) compared to the control and Mn-supplemented groups. The supplemented groups of Mn at 4, 8, and 12 mg kg^−1^ diet exhibited SOD activities similar to the control group in both liver and gill tissues. Interestingly, the gene expression of CAT, SOD, and GPx was also quantified in the present investigation, and the related data are noted in Fig. 3A,B. The CAT (*p* = 0.002), SOD (*p* = 0.0061), and GPx (*p* = 0.014) gene expressions were substantially upregulated with concurrent exposure to arsenic, ammonia, and high-temperature stress, followed by other stressor groups, in comparison to the control and Mn-supplemented groups. Furthermore, the gene expressions of CAT, SOD, and GPx were noticeably downregulated by Mn at 8 mg kg^−1^ diet with stressors (As + NH_3_ + T), followed by the same diet group without stressors, Mn at 4 mg kg^−1^ diet, compared to the control and other treatment groups.

**Table 5:** Effect of dietary manganese (Mn) on CAT, SOD, GST and GPx enzymatic activities against multiple stressors in fish.

Exposure/diets (mg kg^−1^)	Catalase (CAT)		Super-oxide dismutase (SOD)		Glutathione-s-transferase (GST)		Glutathione peroxidase (GPx)	
	CAT-L	CAT-G	SOD-L	SOD-G	GST-L	GST-G	GPx-L	GPx-G
Ctr/Ctr	9.67c ± 0.38	7.54b ± 0.60	45.22a ± 0.97	35.25a ± 1.44	0.32b ± 0.02	0.34b ± 0.04	0.55c ± 0.06	0.44b ± 0.02
As/Ctr	16.95d ± 0.36	12.76d ± 1.21	48.63b ± 0.56	40.37b ± 0.33	0.48c ± 0.06	0.49c ± 0.03	0.81d ± 0.14	0.68d ± 0.06
NH_3_/Ctr	17.48d ± 1.28	12.82d ± 0.83	47.02b ± 1.18	40.94b ± 0.61	0.57d ± 0.03	0.48c ± 0.02	0.78d ± 0.08	0.61d ± 0.08
As + NH_3_/Ctr	22.52e ± 0.87	17.73d ± 1.16	51.02c ± 0.34	41.79b ± 0.32	0.70e ± 0.09	0.63d ± 0.04	0.94e ± 0.14	0.73de ± 0.07
NH_3_ + T/Ctr	23.65e ± 0.89	18.90d ± 1.54	48.52b ± 0.27	42.33b ± 1.21	0.59d ± 0.05	0.63d ± 0.03	0.86de ± 0.04	0.79e ± 0.05
As + T + NH_3_/Ctr	31.51f. ± 1.20	22.94e ± 1.47	49.07b ± 0.29	45.08c ± 0.77	0.94f. ± 0.06	0.82e ± 0.05	1.24f. ± 0.15	1.29f. ± 0.07
Ctr/Mn-4	7.38b ± 0.88	5.81b ± 0.56	46.77a ± 1.0	36.18a ± 1.14	0.22a ± 0.02	0.35b ± 0.02	0.42b ± 0.01	0.44b ± 0.06
Ctr/Mn-8	4.60a ± 0.70	3.98a ± 0.23	44.47a ± 0.56	36.07a ± 1.88	0.18a ± 0.01	0.17a ± 0.01	0.34a ± 0.02	0.29a ± 0.02
Ctr/Mn-12	11.44 cd ± 0.57	10.32c ± 1.04	45.11a ± 0.81	36.46a ± 2.22	0.45c ± 0.02	0.36b ± 0.03	0.81d ± 0.11	0.55c ± 0.08
As + T + NH_3/_Mn-4	6.99b ± 0.76	6.59b ± 0.35	44.36a ± 0.87	37.29a ± 1.79	0.35b ± 0.03	0.40bc ± 0.02	0.58c ± 0.07	0.51c ± 0.02
As + T + NH_3/_Mn-8	4.71a ± 0.90	4.13a ± 0.48	45.19a ± 1.01	35.37a ± 0.80	0.18a ± 0.01	0.20a ± 0.01	0.40ab ± 0.03	0.24a ± 0.01
As + T + NH_3_/Mn-12	9.07c ± 0.79	10.95c ± 1.68	44.81a ± 0.92	37.29a ± 0.69	0.49c ± 0.07	0.45c ± 0.02	0.75d ± 0.05	0.56c ± 0.11
*P*_Value	0.0005	0.0001	0.018	0.037	0.0024	0.0047	0.0018	0.0013

Values in the same row with different superscript (a, b, c, d, e, f) differ significantly. Data expressed as Mean ± SE (*n* = 6). CAT, SOD, GST and GPx: Units/mg protein.

### Effect of Mn on inducible nitric oxide synthase (iNOS) and Na^+^K^+^ATPase gene expression

In the present investigation, the inducible nitric oxide synthase (iNOS) in liver tissue was quantified, and the results are presented in Fig. 3B. The iNOS gene expression was remarkably upregulated (*p* = 0.0036) by the As + NH_3_ + T group, followed by arsenic alone, compared to the control and other groups. Exposure to NH_3_, As + NH_3_, and NH_3_ + T groups showed similar iNOS gene expression levels. Dietary supplementation of Mn at 8 mg kg^−1^ group noticeably downregulated iNOS gene expression, with or without stressors, followed by Mn at 4 mg kg^−1^ diet, compared to the control and other treatments. Additionally, the Na^+^K^+^ATPase gene expression was quantified in the present investigation, and the data are presented in Fig. 3C. The gene expression of Na^+^K^+^ATPase was noticeably upregulated (*p* = 0.0022) by As + NH_3_ and As + NH_3_ + T, followed by As alone and NH_3_ + T, compared to the control and other groups. Moreover, Na^+^K^+^ATPase was significantly downregulated with the supplementation of Mn at 8 mg kg^−1^ diet, with or without stressors, compared to the control and other treatment groups.

### Effect of Mn on growth performance attributes and gene regulation

**Figure 4:**
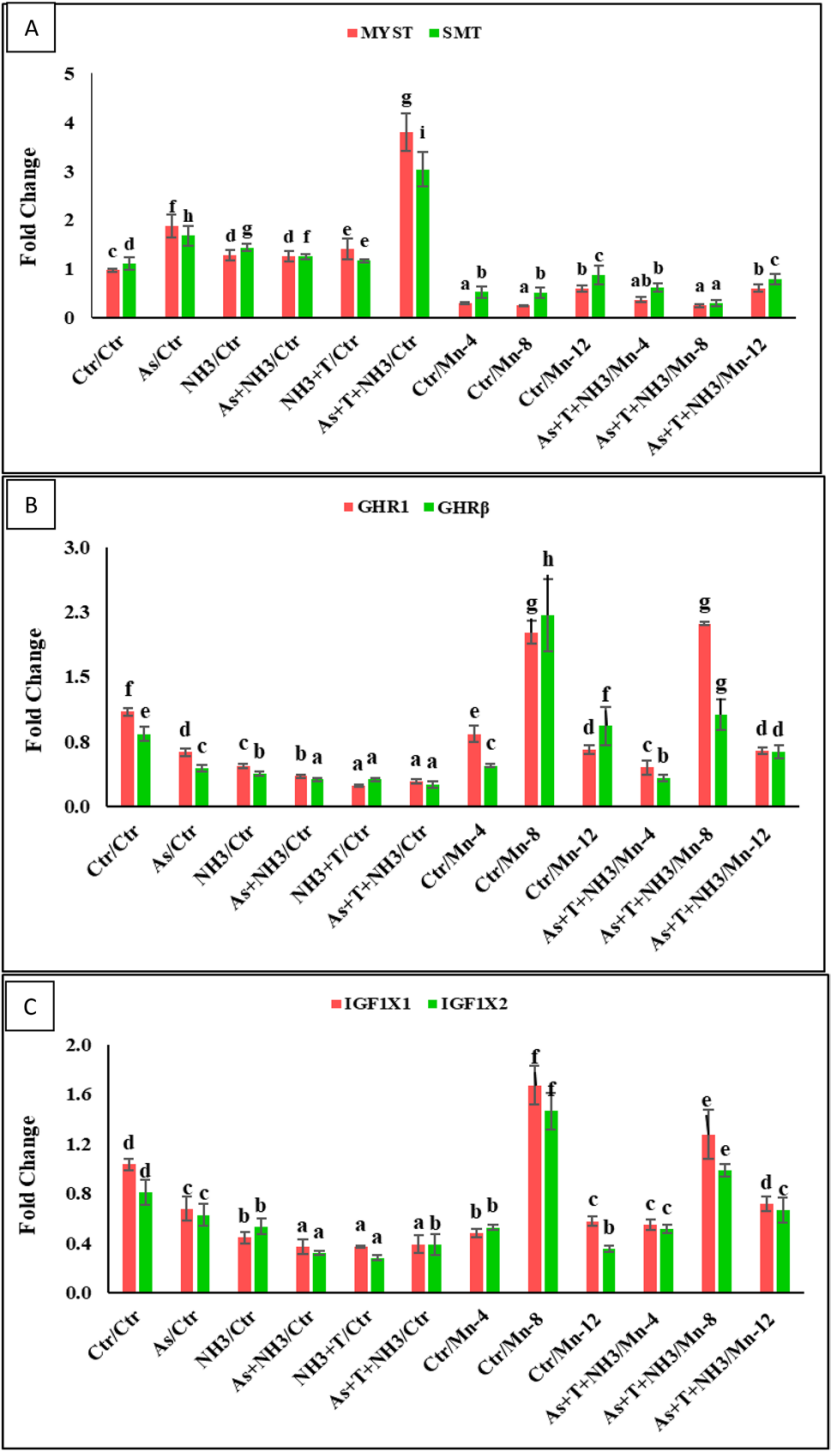
Manganese diets regulate the gene expression of MYST, SMT, GHR1, GHRβ, IGF1X1, and IGF1X2 against multiple stressors in fish. Within endpoints and groups, bars with different superscripts differ significantly (a–g) MYST *p* = 0.0023), SMT *p* = 0.0042), GHR1 *p* = 0.0027), GHRβ *p* = 0.0033), IGF1X1 *p* = 0.015), IGD1X2 *p* = 0.0072). Data expressed as Mean ± SE (*n* = 3).

In the present investigation, genes related to growth performance, such as growth hormone (GH), myostatin (MYST), somatostatin (SMT), growth hormone regulatory (GHR1 and GHRβ), and insulin-like growth factors (IGF1X1 and IGF1X2), were quantified, and the data are presented in Figs. 3C and 4A-C. GH was significantly upregulated (*p* = 0.0016) by the supplementation of Mn at 8 mg kg^−1^ diet, with or without stressors, compared to the control and other treatment groups, including other Mn-supplemented diets. Moreover, GH gene regulation was noticeably downregulated by As + NH_3_ + T, As + NH_3_, and NH_3_ + T, in comparison to the control and supplemented groups. Furthermore, Mn at 4 and 12 mg kg^−1^ in the diet did not effectively regulate GH gene expression (Fig. 3C). On the other hand, MYST (*p* = 0.0023) and SMT (*p* = 0.0042) gene regulations were remarkably upregulated by concurrent exposure to the As + NH_3_ + T stress group, in comparison to the control and other treatment groups. Moreover, dietary supplementation of Mn at 8 mg kg^−1^ diet significantly downregulated MYST and SMT in the liver tissue of *P. hypophthalmus*, compared to the control and other treatment groups (Fig. 4A). Surprisingly, GHR1 (*p* = 0.0027), GHRβ (*p* = 0.0033), IGF1X1 (*p* = 0.015), and IGF1X2 (*p* = 0.0072) genes were substantially upregulated with the supplementation of Mn at 8 mg kg^−1^ in the diet, with or without stressors (As + NH_3_ + T), followed by Mn at 4 mg kg^−1^ in the diet, in comparison to the control and other treatment groups. In contrast, GHR1, GHRβ, IGF1X1, and IGF1X2 genes were significantly downregulated by stressors (As + NH_3_ + T, As + NH_3_, NH_3_ + T, As, and NH_3_), compared to the control and other treatment groups (Fig. 4 B,C).

In the present investigation, the growth performance indicators of *P. hypophthalmus* viz. final weight gain %, FCR, SGR, PER, DGI, TGC and RFI were presented in Table 6. Results of final weight gain and SGR, PER, DGI and RFI were significantly reduced with concurrent exposure to arsenic, ammonia, and high-temperature stress, followed by NH_3_ + T, respectively As + NH_3_, NH_3_, and As groups compared to control and Mn-supplemented groups. Further, the supplementation of Mn at 8 mg kg^−1^ diet with or without stressors was noticeably enhanced, followed by Mn at 4 mg kg^−1^ diet compared to control and other treatments. Whereas, the results of FCR were significantly inverse to SGR and PER. The Mn diet at 8 mg kg^−1^ diet was observed significantly lowest FCR followed by Mn at 4 mg kg^−1^ diet with or without stressors. However, the highest FCR was observed in the concurrent exposure to As + NH_3_ + T and control fed group.

**Table 6:** Effect of dietary manganese (Mn) on growth performance viz. final body weight gain (%), FCR, SGR, PER, DGI (%), TGC and RFI against multiple stressors in fish.

Exposure/diets (mg kg^−1^)	Final body weight gain %	FCR	SGR	PER	DGI (%)	TGC	RFI
Ctr/Ctr	95.20d ± 3.26	3.22c ± 0.09	0.57c ± 0.01	0.89d ± 0.02	1.00de ± 0.03	0.0396	305.58d ± 2.38
As/Ctr	51.83b ± 3.27	5.25d ± 0.28	0.33b ± 0.02	0.55b ± 0.05	0.58b ± 0.02	0.0404	270.46b ± 2.52
NH_3_/Ctr	49.89b ± 0.63	5.40d ± 0.06	0.35b ± 0.01	0.53b ± 0.02	0.56b ± 0.01	0.0389	269.22b ± 0.48
As + NH_3_/Ctr	50.25b ± 3.67	5.39d ± 0.33	0.34b ± 0.01	0.55b ± 0.05	0.56b ± 0.03	0.0390	268.47b ± 2.56
NH_3_ + T/Ctr	49.91b ± 0.54	5.36e ± 0.05	0.35b ± 0.02	0.58b ± 0.01	0.56b ± 0.01	0.0308	267.28b ± 2.11
As + T + NH_3_/Ctr	42.03a ± 0.77	6.16f. ± 0.09	0.27a ± 0.03	0.50a ± 0.01	0.48a ± 0.02	0.0306	258.66a ± 0.91
Ctr/Mn-4	152.16f. ± 3.48	2.27b ± 0.04	0.86d ± 0.02	1.37f. ± 0.02	1.40f. ± 0.02	0.0391	344.68f. ± 2.36
Ctr/Mn-8	191.60 g ± 6.10	2.06a ± 0.06	1.08e ± 0.01	1.57 h ± 0.04	1.66 g ± 0.05	0.0382	394.17 h ± 1.78
Ctr/Mn-12	75.54c ± 2.69	3.87c ± 0.12	0.57c ± 0.02	0.74c ± 0.03	0.82c ± 0.03	0.0400	291.94c ± 1.84
As + T + NH_3/_Mn-4	136.94e ± 4.85	2.42b ± 0.06	0.82d ± 0.03	1.21e ± 0.01	1.33e ± 0.03	0.0305	331.24e ± 3.92
As + T + NH_3/_Mn-8	197.42 g ± 2.38	1.98a ± 0.03	1.11e ± 0.04	1.49 g ± 0.05	1.72 h ± 0.01	0.0308	389.88 g ± 2.59
As + T + NH_3_/Mn-12	86.73c ± 5.91	3.44 ± c0.23	0.60c ± 0.03	0.93d ± 0.07	0.92d ± 0.05	0.0308	295.46c ± 1.87
P-Value							

Values in the same row with different superscript (a, b, c, d, e) differ significantly. Data expressed as Mean ± SE (*n* = 3). FCR: feed conversion ratio; SGR: specific growth rate; PER: protein efficiency ratio; DGI: Daily growth index; TGC: Thermal growth coefficient; RFI: relative feed intake.

### Effect of Mn on LPO, Vit C and haematological parameters

The results of lipid peroxidation (LPO) in the liver and kidney, and Vitamin C levels in muscle and brain, as well as the counts of RBC, WBC, and Hb in *P. hypophthalmus* reared under control conditions and multiple stressors (As, NH_3_, As + NH_3_, NH_3_ + T, As + NH_3_ + T), and fed with control and Mn-supplemented diets, are presented in Table 7. LPO levels in the liver (*p* = 0.0022) and kidney (*p* = 0.0053) were significantly higher with concurrent exposure to ammonia, arsenic, and high temperature, followed by other stressors, compared to control and Mn-supplemented diets. Interestingly, Mn at 8 mg kg^−1^ diet, with or without stressors, significantly reduced LPO levels in the liver and kidney, followed by Mn at 4 mg kg^−1^ diet, compared to the control and other treatment groups. Vitamin C levels in muscle (*p* = 0.015) and brain (*p* = 0.021) were noticeably elevated with dietary Mn at 8 mg kg^−1^ diet, with or without stressors, compared to control and other treatment groups. Further, Vitamin C levels in muscle and brain were significantly lowered with concurrent exposure to arsenic, ammonia, and high temperature, followed by other stressor groups, compared to control and Mn-supplemented groups. Surprisingly, total blood counts such as RBC and Hb were significantly elevated with stressors (As + NH_3_ + T, As + T, As + NH_3_, NH_3_, and As) compared to control and Mn-supplemented groups. Moreover, RBC (*p* = 0.0066) and Hb (*p* = 0.017) counts were noticeably reduced with dietary Mn at 8 mg kg^−1^ diet, followed by Mn at 4 mg kg^−1^ diet, with or without stressors, in comparison to control and other treatment groups. In contrast, WBC counts (*p* = 0.019) were inversely related to RBC and Hb, as they were significantly reduced with different stressors such as arsenic, ammonia, and high temperature. Moreover, Mn supplementation considerably enhanced WBC counts (Mn at 8 and 4 mg kg^−1^ diet).

**Table 7:** Effect of dietary manganese (Mn) on LPO, Vit C, RBC, WBC and Hb against multiple stress in fish.

Exposure/diets (mg kg^−1^)	LPO-L	LPO-K	Vit C-M	Vit C-B	RBC	WBC	Hb
Ctr/Ctr	1.94c ± 0.44	20.77b ± 0.54	18.94e ± 0.54	16.50d ± 0.77	1.29c ± 0.0058	66.21e ± 0.90	5.09b ± 0.15
As/Ctr	2.93e ± 0.46	25.91c ± 0.37	12.82c ± 0.75	10.99c ± 0.72	1.36d ± 0.011	54.96c ± 0.99	5.78c ± 0.10
NH_3_/Ctr	3.31ef ± 0.82	25.39c ± 1.96	12.73c ± 1.0	11.25c ± 1.03	1.34c ± 0.0053	54.43c ± 1.87	5.69c ± 0.03
As + NH_3_/Ctr	3.25ef ± 0.41	32.34d ± 1.38	10.68b ± 0.76	8.24b ± 0.52	1.39e ± 0.051	50.69b ± 0.73	6.11d ± 0.15
NH_3_ + T/Ctr	4.77f. ± 0.38	30.24d ± 0.37	9.65b ± 0.45	12.41c ± 3.28	1.40e ± 0.0087	50.19b ± 1.16	5.99c ± 0.25
As + T + NH_3_/Ctr	5.46 g ± 0.55	40.89e ± 0.86	7.48a ± 0.52	5.79a ± 0.33	1.46f. ± 0.0084	45.98a ± 0.85	6.39e ± 0.02
Ctr/Mn-4	1.86c ± 0.47	20.94b ± 1.48	19.62e ± 0.57	16.22d ± 0.56	1.28b ± 0.047	65.02e ± 0.41	4.97a ± 0.14
Ctr/Mn-8	0.94a ± 0.02	12.77a ± 0.52	26.31 g ± 0.87	23.68f. ± 0.89	1.22a ± 0.0076	72.43f. ± 1.58	4.65a ± 0.03
Ctr/Mn-12	2.35 cd ± 0.28	24.82c ± 0.59	14.34d ± 0.90	9.88c ± 0.78	1.32c ± 0.011	57.42d ± 0.80	5.19b ± 0.15
As + T + NH_3/_Mn-4	1.79bc ± 0.056	20.26b ± 0.97	18.69e ± 0.78	15.80d ± 0.66	1.31c ± 0.008	60.82e ± 1.09	4.82a ± 0.08
As + T + NH_3/_Mn-8	1.27b ± 0.02	13.23a ± 1.02	23.89f. ± 1.03	21.15e ± 0.69	1.20a ± 0.02	71.03f. ± 0.87	4.65a ± 0.05
As + T + NH_3_/Mn-12	2.78d ± 0.025	20.76b ± 0.62	14.59d ± 0.56	16.50d ± 0.15	1.33c ± 0.01	58.93d ± 1.08	5.20b ± 0.17
*P*_Value	0.0022	0.0053	0.015	0.021	0.0066	0.019	0.017

Values in the same row with different superscript (a, b, c, d, e, f) differ significantly. Data expressed as Mean ± SE (*n* = 6). LPO: n mole TBARS formed/h/mg protein; Vit C: µg/g of wet tissue; RBC: Number (10^6^ cell/mm^3^); WBC: Number (10^3^ Cell/mm^3^); Hb: gm (dl).

#### Effect of Mn on immunological attributes

The data on immunological attributes, such as nitroblue tetrazolium (NBT), blood glucose (BG), total protein, albumin, globulin, A:G ratio, and myeloperoxidase (MPO) in *P. hypophthalmus* reared under arsenic, ammonia, and high temperature, and fed with different levels of Mn, are presented in Table 8. The levels of NBT (*p* = 0.018), total protein (*p* = 0.023), and globulin (*p* = 0.011) were noticeably inhibited with concurrent exposure to ammonia, arsenic, and high-temperature groups, in comparison to all other groups. However, NBT was significantly reduced in the ammonia and high-temperature groups. Further, Mn at 8 mg kg^−1^ diet was noticeably elevated, with supplementation of Mn at 8 mg kg^−1^ diet, followed by other Mn-supplemented groups, in comparison to the control and other treatment groups. Whereas BG (*p* = 0.0046) and A:G ratio (*p* = 0.029) were significantly reduced with supplementation of Mn at 8 mg kg^−1^ diet, compared to the control, Mn at 4 and 12 mg kg^−1^ diet, and stressor groups. Similarly, levels of MPO were significantly elevated with Mn at 8 mg kg^−1^ diet, with or without stressors, followed by Mn at 4 mg kg^−1^ diet, compared to the control and other treatment groups.

**Table 8:** Effect of dietary manganese (Mn) on NBT, BG, total protein, albumin, globulin, A:G ratio, Ig and MPO against multiple stressors in fish.

Exposure/diets (mg kg^−1^)	NBT	BG	Total Protein	Albumin	Globulin	A:G ratio	MPO
Ctr/Ctr	0.56f. ± 0.05	111.23c ± 5.44	0.76e ± 0.03	0.26 ± 0.003	0.71e ± 0.12	0.36b ± 0.03	0.29 ± 0.003
As/Ctr	0.21ab ± 0.01	129.75d ± 3.98	0.55d ± 0.05	0.20 ± 0.035	0.34b ± 0.02	0.58d ± 0.09	0.19a ± 0.006
NH_3_/Ctr	0.24b ± 0.03	140.86e ± 1.69	0.50c ± 0.03	0.16 ± 0.017	0.35b ± 0.01	0.45c ± 0.02	0.18a ± 0.003
As + NH_3_/Ctr	0.20a ± 0.01	144.66f. ± 4.18	0.39b ± 0.03	0.15 ± 0.012	0.25a ± 0.02	0.60e ± 0.06	0.17a ± 0.007
NH_3_ + T/Ctr	0.18a ± 0.02	168.93 g ± 6.17	0.54d ± 0.04	0.16 ± 0.006	0.38b ± 0.03	0.44c ± 0.03	0.16a ± 0.001
As + T + NH_3_/Ctr	0.25b ± 0.03	146.61f. ± 5.0	0.32a ± 0.03	0.11 ± 0.002	0.21a ± 0.01	0.54d ± 0.03	0.16a ± 0.003
Ctr/Mn-4	0.48d ± 0.02	110.37c ± 5.97	0.81f. ± 0.04	0.19 ± 0.001	0.62d ± 0.03	0.30b ± 0.01	0.32c ± 0.038
Ctr/Mn-8	0.56e ± 0.06	88.05a ± 2.67	1.05 g ± 0.04	0.14 ± 0.009	0.91 g ± 0.05	0.15a ± 0.00	0.38d ± 0.009
Ctr/Mn-12	0.32c ± 0.02	129.75d ± 3.30	0.46bc ± 0.03	0.13 ± 0.014	0.33b ± 0.05	0.40b ± 0.09	0.20ab ± 0.006
As + T + NH_3/_Mn-4	0.44d ± 0.07	112.13c ± 6.71	0.76e ± 0.06	0.23 ± 0.056	0.53d ± 0.03	0.47c ± 0.02	0.27b ± 0.007
As + T + NH_3/_Mn-8	0.53e ± 0.03	95.13b ± 2.32	1.02f. ± 0.08	0.18 ± 0.014	0.84f. ± 0.03	0.21a ± 0.02	0.38d ± 0.006
As + T + NH_3_/Mn-12	0.33c ± 0.02	129.80d ± 4.15	0.60de ± 0.03	0.15 ± 0.017	0.45c ± 0.05	0.34b ± 0.05	0.22a ± 0.007
*P*_Value				0.071			

Values in the same row with different superscript (a, b, c, d, e) differ significantly. Total protein, albumin, globulin: g dL^−1^Blood glucose: mgdL^−1^; Data expressed as Mean ± SE (*n* = 3).

#### Effect of Mn on protein and carbohydrate metabolic enzymes

In the present investigation, the data on ALT, AST, LDH, and MDH activities in the liver and gill, as well as acetylcholine esterase (AChE) in the brain of *P. hypophthalmus*, are recorded in Table 9. ALT and AST activities in the liver and gill were noticeably elevated (*p* < 0.01) with concurrent exposure to As, NH_3_, and high temperature, followed by As + NH_3_, NH_3_ + T, and other stressor groups, compared to the control and Mn-supplemented groups. Moreover, ALT, AST, LDH, and MDH activities in the liver and gill were remarkably reduced with Mn at 8 mg kg^−1^ diet, with or without stressors, followed by Mn at 4 mg kg^−1^ diet, compared to the control and other treatment groups. Furthermore, Mn at 12 mg kg^−1^ diet was not effective in modulating the activities of ALT, AST, LDH, and MDH against multiple stressors.

**Table 9:** Effect of dietary manganese (Mn) on ALT, AST, LDH, MDH and AChE enzymatic activities against multiple stressors in fish.

Exposure/diets (mg kg^−1^)	ALT-L	ALT-G	AST-L	AST-G	LDH-L	LDH-G	MDH-L	MDH-G	AChE
Ctr/Ctr	11.66c ± 1.11	5.43b ± 0.49	11.86 cd ± 0.95	14.26d ± 1.26	1.94b ± 0.23	5.40d ± 0.37	0.97b ± 0.04	1.23c ± 0.03	0.53d ± 0.04
As/Ctr	14.49d ± 1.92	8.04c ± 0.57	16.14e ± 1.03	22.81e ± 1.67	2.93bc ± 0.18	8.15e ± 0.31	1.19c ± 0.02	1.79d ± 0.07	0.48c ± 0.06
NH_3_/Ctr	14.87d ± 1.20	9.57c ± 1.31	17.54e ± 0.55	23.38e ± 2.43	3.31c ± 0.26	8.57e ± 0.40	1.55c ± 0.05	1.69d ± 0.04	0.43bc ± 0.05
As + NH_3_/Ctr	16.08e ± 2.27	10.60d ± 1.11	19.75f. ± 1.34	23.44e ± 2.46	3.25c ± 0.11	7.82e ± 0.24	1.16c ± 0.06	1.52d ± 0.08	0.38b ± 0.04
NH_3_ + T/Ctr	16.69e ± 0.95	14.30e ± 1.62	19.42f. ± 1.77	25.21f. ± 2.38	4.77d ± 0.27	7.34e ± 0.23	1.31c ± 0.09	1.73d ± 0.14	0.39b ± 0.03
As + T + NH_3_/Ctr	20.90f. ± 1.71	18.87f. ± 2.21	22.58 g ± 2.09	31.26 g ± 1.70	5.46e ± 0.18	11.06f. ± 0.34	1.81d ± 0.06	2.05e ± 0.12	0.33a ± 0.04
Ctr/Mn-4	9.07b ± 0.92	6.47b ± 1.23	7.91b ± 0.58	12.19c ± 1.63	1.86ab ± 0.15	3.79c ± 0.31	0.93b ± 0.03	0.82b ± 0.014	0.53d ± 0.02
Ctr/Mn-8	5.76a ± 0.53	3.72a ± 0.78	4.87a ± 0.70	7.57a ± 0.76	0.94a ± 0.11	1.66a ± 0.91	0.62ab ± 0.15	0.57a ± 0.04	0.69e ± 0.07
Ctr/Mn-12	14.14d ± 0.93	14.76e ± 1.14	13.09d ± 1.46	12.78c ± 1.15	2.35bc ± 0.28	4.86 ± 0.83	1.29d ± 0.04	1.28c ± 0.13	0.39b ± 0.05
As + T + NH_3/_Mn-4	8.39b ± 0.80	5.95b ± 0.52	9.32c ± 1.04	10.25b ± 0.73	1.79b ± 0.16	2.60b ± 0.78	0.94b ± 0.04	1.05bc ± 0.12	0.51d ± 0.05
As + T + NH_3/_Mn-8	5.62a ± 0.43	3.07a ± 1.15	6.09b ± 0.37	7.88a ± 0.90	1.27a ± 0.15	1.54a ± 0.27	0.42a ± 0.01	0.68a ± 0.04	0.72e ± 0.08
As + T + NH_3_/Mn-12	14.85d ± 1.24	11.12d ± 1.06	10.13c ± 0.76	13.25 cd ± 0.76	2.78bc ± 0.36	3.67c ± 0.87	1.09c ± 0.03	1.65d ± 0.03	0.44bc ± 0.06
*P*_Value	0.0054	0.018	0.0072	0.0081	0.003	0.0063	0.0029	0.0015	0.0039

Values in the same row with different superscript (a, b, c, d, e, f) differ significantly. Data expressed as Mean ± SE (*n* = 6). ALT: nmole of sodium pyruvate formed/mg protein/min at 37 °C, AST: nmole oxaloacetate released/min/mg protein at 37 °C. LDH and MDH: units/min/mg protein at 37 °C, AChE: nmole/min/mg protein.

#### Effect of Mn on neurotransmitter

Interestingly, the AChE activities in the brain were remarkably inhibited (*p* = 0.0039) with concurrent exposure to arsenic, ammonia, and high temperature, followed by NH_3_ + T, As + NH_3_, NH_3_, and As groups, in comparison to the control and Mn-supplemented groups. Conversely, AChE activities were noticeably elevated with Mn at 8 mg kg^−1^ in the diet, compared to the control, Mn at 4 and 12 mg kg^−1^ in the diet, and stressor groups (Table 9).

#### Effect of Mn on bioaccumulation of arsenic

The results of bioaccumulation and concentration of arsenic in different fish tissues and experimental water are presented in Table 10. The arsenic concentration in water was determined to be the highest in the group treated under arsenic, ammonia, and high temperature and fed with a control diet (1776 µg L^−1^), followed by Mn at 12 mg kg^−1^ diet with stressors (As + NH_3_ + T) (1337 µg L^−1^), the arsenic alone group (1186 µg L^−1^), and Mn at 4 mg kg^−1^ diet with stressors (1040 µg L^−1^) groups. Meanwhile, the bioaccumulation of arsenic was found to be highest in the liver and kidney tissues treated under As + NH_3_ + T. Arsenic was below the detection limit in the groups treated with Mn at 4 and 8 mg kg^−1^ diet, as well as in the control groups, in muscle and brain tissues. Moreover, in the same diets but exposed to As + NH_3_ + T, the arsenic concentration was the least in muscle and brain tissues. Furthermore, Mn bioaccumulation was highest in the Mn-12 mg kg^−1^ diet, followed by Mn at 4 and 8 mg kg^−1^ in the diet.

**Table 10:** Effect of dietary manganese (Mn) on detoxification of arsenic in different fish tissues reared under control and multiple stressors condition.

Exposure/diets (mg kg^−1^)	Water (µg L^−1^)	Liver (mg kg^−1^)	Kidney (mg kg^−1^)	Gill (mg kg^−1^)	Muscle (mg kg^−1^)	Brain (mg kg^−1^)	Mn-Muscle (mg kg^−1^)
Ctr/Ctr	0.06 ± 0.001	0.01 ± 0.001	0.04 ± 0.0001	0.04 ± 0.001	BDL	BDL	0.42 ± 0.02
As/Ctr	1077.51 ± 33.37	5.24 ± 0.15	5.95 ± 0.28	3.21 ± 0.05	1.57 ± 0.11	0.33 ± 0.046	0.64 ± 0.07
NH_3_/Ctr	0.04 ± 0.001	0.03 ± 0.01	0.11 ± 0.01	0.02 ± 0.001	0.04 ± 0.01	BDL	0.80 ± 0.12
As + NH_3_/Ctr	1186.28 ± 21.09	5.84 ± 0.09	6.60 ± 0.17	3.79 ± 0.07	1.42 ± 0.13	0.36 ± 0.038	0.36 ± 0.06
NH_3_ + T/Ctr	0.09 ± 0.01	0.04 ± 0.001	0.07 ± 0.01	0.08 ± 0.001	0.02 ± 0.001	BDL	0.47 ± 0.04
As + T + NH_3_/Ctr	1776.47 ± 55.13	7.40 ± 0.18	7.08 ± 0.07	1.76 ± 0.05	2.08 ± 0.06	0.49 ± 0.019	0.33 ± 0.04
Ctr/Mn-4	0.03 ± 0.01	0.01 ± 0.001	0.07 ± 0.01	0.09 ± 0.001	BDL	BDL	5.74 ± 0.20
Ctr/Mn-8	0.03 ± 0.001	BDL	0.05 ± 0.001	0.02 ± 0.001	BDL	BDL	9.49 ± 0.15
Ctr/Mn-12	0.03 ± 0.001	0.03 ± 0.001	0.09 ± 0.002	0.07 ± 0.01	0.04 ± 0.001	0.05 ± 0.004	14.17 ± 0.14
As + T + NH_3/_Mn-4	1040.62 ± 28.97	2.99 ± 0.13	3.54 ± 0.14	3.03 ± 0.10	0.74 ± 0.11	0.73 ± 0.022	3.22 ± 0.05
As + T + NH_3/_Mn-8	243.50 ± 6.89	0.37 ± 0.03	0.57 ± 0.06	0.80 ± 0.07	0.17 ± 0.07	0.06 ± 0.008	6.48 ± 0.54
As + T + NH_3_/Mn-12	1337.87 ± 49.84	2.85 ± 0.11	2.17 ± 0.04	2.89 ± 0.32	1.67 ± 0.30	0.81 ± 0.01	11.18 ± 0.45

#### Effect of Mn on bacterial infection

**Figure 5:**
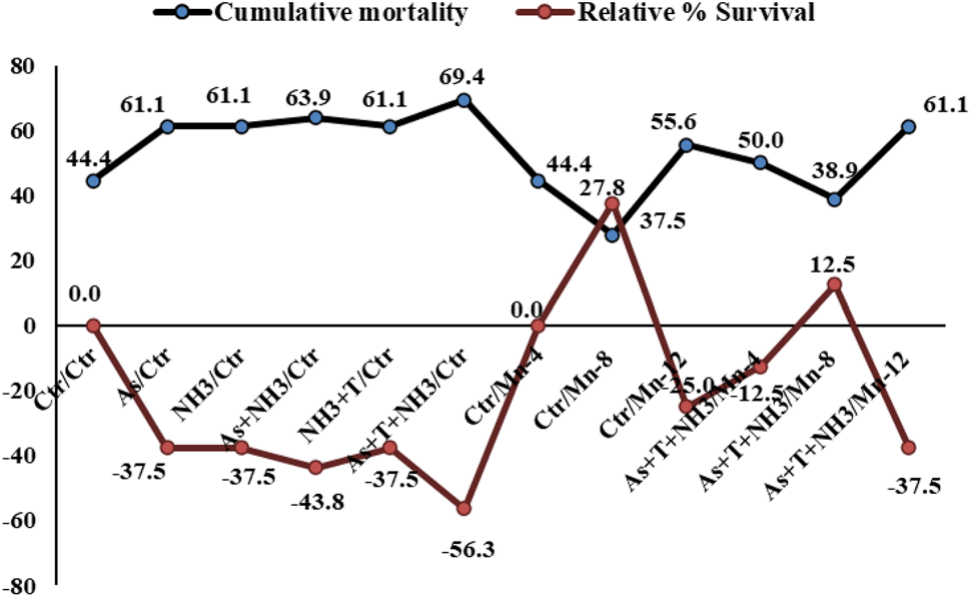
Manganese diets reduces cumulative mortality and enhances relative percentage survival against pathogenic bacteria in fish reared under multiple stressors.

After the experiment, the fish were infected with the *Aeromonas hydrophila*. Cumulative and relative % survival was determined up to seven days after the fish were infected. Cumulative mortality was observed to be higher in the group treated with concurrent exposure to As, NH_3_, and T, followed by As + NH_3_ (63), NH_3_ + T (61), and Mn at 12 mg kg^−1^ in the diet with stressors (61). In contrast, the least mortality was observed in Mn at 8 mg kg^−1^ in the diet (27) with stressors (38). Similarly, the relative % survival was observed as -37.5, -37.5, -48.3, -37.5, -56, 0, 27, -25, -12.5, 12.5, -37% for As, NH_3_, As + NH_3_, NH_3_ + T, As + NH_3_ + T, Mn at 4, 8, and 12 mg kg^−1^ in the diet, and Mn at 4, 8, and 12 mg kg^−1^ diet with stressors, respectively (Fig. 5).

## Discussion

Cortisol is secreted from the inter-renal cell of head kidney released directly into the blood^55^. It is an important stress hormone plays a role in growth, reproduction, and osmoregulation^56^. The multiple stressors (As + NH_3_ + T) induces stress in the fish which needed energy to combat the stress which compensated through glucogenic pathway. The stress indues by As + NH_3_ + T, that elevated cortisol levels could be due to the stressors targeting the multiple sites in the hypothalamus-pituitary-interrenal axis and altered the adrenocorticotropic hormone (ACTH) secretion^57–59^. Moreover, the dietary Mn at 8 mg kg^−1^ diet followed by 4 mg kg^−1^ diet noticeably reduced the cortisol levels which could be due to Mn support the energy provided to glucogenic pathway^60^. During this stage the physiological change also occurred and adapted by fish and homeostasis return which was supported by Mn diet.

HSP 70 are chaperones protein, maintain the normal structure and function of cell protein and help in folding protein into unfolded protein^61^ and indicating physiological conditions of fish during stress. HSPs protein expression is generally upregulated during temperature and metal stress^62,63^. Indeed, the Mn-containing diet downregulated the HSP 70 expression might be due to its role in ApoA-1 gene regulation. It is also proven that if brain could not control oxidative challenges, heat shock protein upregulates the HSPs during stress. The present study showed that Mn at 8 mg kg^−1^ diet helps in maintenance of the cellular homeostasis through correct folding of nascent and stress-accumulated misfolded proteins in the cell^64^. This might be due to activating the transcription factor of HSP by Mn as it loses the binding activity of heat shock elements, and thus Mn downregulates the HSP70 expression^65,66^.

The cytochrome P450 is the heme-thiolate protein, is a major component of the membrane-bound microsomal monooxygenase system (MMO), which helps in catalyzes the oxygenation of exogenous and endogenous compounds (Xenobiotics, drugs, and carcinogens)^67^. Moreover, the toxicity of arsenic, ammonia, and high-temperature stress upregulated the CYP 450 gene expression as it generates ROS, which potentially causes lipid peroxidation, cell toxicity, and death. Interestingly, it is inferred that CYP 450 involved in the arsenic, ammonia toxicity and high temperature stress which take part for apoptosis and upregulated the transcription of bcl2-associated X (Bax)^68^. Bax is the important cell death promoting gene in fish which induce release of cytochrome c, leading to caspase activation^69^. Surprisingly, Mn at 8 mg kg^−1^ diet was remarkably downregulated the CYP 450 gene expression might be due to it regulating and controlling the generated reactive oxygen systems (ROS), cytokines regulation, and lipid peroxidation. In the present study, results of ROS, LPO, and cytokines regulation supported the role of Mn in the control CYP 450 regulation.

DNA damage and DNA damage inducible protein (DDIP) gene expression was upregulated by ammonia, arsenic and high temperature stress could be due to extensive generation of reactive oxygen species, dysregulation of cell proliferation, apoptosis, diminished DNA repair, aberrant in histone post-translational modification and DNA methylation^70^. However, the dietary Mn at 8 mg kg^−1^ diet protect against DNA damage and downregulated DDIP might be due to at this lower dose of Mn enhances the viability of SH-SY5Y cells, reduced the ROS production and LPO levels as well as enhances GSH levels^71^. Interestingly, the MT gene expression was highly upregulated by arsenic, ammonia, and high temperature stress and downregulated by Mn diet. Moreover, the higher dietary Mn induces overexpression of MT gene^72^.

Apoptosis indicates cell programming death in which Cas 3a and 3b belong to the apoptosis gene. The Cas 3a and 3b were upregulated due to arsenic, ammonia, and high-temperature stress could incur apoptosis using p53 and regulated the apoptosis in the liver tissue^73^ and upregulated gene related to oxidative stress and inflammatory response as shown in the present study. Indeed, the supplementation of Mn at 8 and 4 mg kg^−1^ diet help in controlling the regulation of Cas 3a and 3b, which might be due to it has a role in the activation of the caspase cascade and DNA fragmentation in the liver cell^74^.

The present study revealed that stressors (As + NH_3_ + T) reduced the immunity of the fish through cytokines gene upregulation such as TNFα, TLR and IL and downregulated the Ig gene. Zhang et al^75^. reported that the ammonia toxicity altered the immunity of the fish. The stressors induced the stress and showed higher inflammation rate in liver tissue in fish and hence the higher upregulation of TNFα, IL and TLR was determined in the present study^2^. The TNFα, IL and TLR acts as an essential pro-inflammatory cytokine that enhances the immunity in aquatic animals including fish^76^. Notably, the Mn at 8 mg kg^−1^ diet was improved the immunity of the fish using the strengthening/downregulating the TNFα, IL and TLR. This might be due to Mn have an important role in immunostimulants and activating the NF-κB signaling pathways to enhance immunity of the fish against multiple stresses. In contrast to the results of TNFα, IL, TLR and Ig was downregulated with stressors (As + NH_3_ + T, NH_3_ + T, As + NH_3_, NH_3_ and As) and upregulated by Mn diet. This could be due to the role of Mn in enhancing humoral and cell-mediated immunity and improving antibody affinity, early β cell development, complement system, cell mediated immunity, phagocytose activity, and antibody reaction.

The present study pointed out the remarkable reduction of oxidative stress enzymes (SOD, CAT, GST, and GPx activities) and gene expression of SOD, CAT, and GPx through Mn diet at 8 mg kg^−1^. This could be due to the role of manganese in substituting as a cofactor for iron in certain enzymes which is responsible for oxidative stress elevation^77^. Mn is also a cofactor for many enzymes, including pyruvate carboxylase and manganese superoxide dismutase (Mn-SOD). It also protects the cell against reactive oxygen species (ROS) as Mn is part of metalloenzyme by catalyzing the one-electron reduction of peroxide anion to hydrogen peroxide^78^. Mn is found in the Mn-SOD complex, which is useful in maintaining the structure of antioxidant enzymes^79,80^, affecting the Fenton reaction. Mn also enhances the organism's anti-oxidative status through synthesizing and activating certain enzymes such as oxidoreductase transferase, hydrolases, and ligase, as well as vitamins C and B. It also involved metalloenzymes such as arginase, glutamine synthetase, phosphoenol pyruvate, and decarboxylase^81^. Moreover, Mn is the essential mineral nutrient for managing aquatic animals' oxidative stress.

Interestingly, iNOS gene expression was notably highly upregulated by stressors (As + NH_3_ + T, NH_3_ + T, As + NH_3_, NH_3_ and As) could be due to higher accumulation of NH_3_ in fish tissues. Moreover, the NH_3_ is converted into urea via ornithine-urea cycle (OUC) and then converted into glutamine via the glutamine synthetase including non-essential amino acids^82^. Similarly, the blood carrying the high ammonia concentration and affecting the liver tissue^83^. Moreover, nitric oxide provided protection to cellular system against oxidative stress^84^. Moreover, the dietary Mn at 8 mg kg^−1^ diet remarkably downregulated the iNOS gene expression in liver tissue. Further, during stress condition, the organism needs more energy in the form of ATPase, therefore the gene expression of Na^+^K^+^ATPase was highly upregulated. Notably, the Mn diet help in formation of more ATPase and supplied to the fish reread under multiple stress condition.

The growth performance related gene expression viz. GH, GHR1, GHRβ, IGF1X1 and IGF1X2 were remarkably downregulated by stressors (As + NH_3_ + T, NH_3_ + T, As + NH_3_, NH_3_ and As) could be due to disruption of endocrine receptor which control the growth related gene expression. The GH gene bind with GHR and controlled by hypothalamic regulation viz. GH-releasing hormone, ghrelin, dopamine and somatostatin^85,86^. The growth-related genes mainly regulated by genetically, endocrinologically and environmentally. It is also related with better nutrition, optimum temperature, good husbandry condition and better functioning of endocrine regulation^87^. It is also observed that the Mn diet notably downregulated MYST and SMT at 8 mg kg^−1^ diet. It might be due to the role of MYST in decreasing the myoblast, which results in terminal differentiation and division of fiber enlargement^88^. Further, the IGF1X1 and IGF1X2 gene expressions have important role in biomolecular regulation such as carbohydrates, lipid, protein, and mineral metabolism, differentiation and proliferation of the cell and ultimately growth^89^. As the GH bind to the receptor in the liver cell to stimulate, release and synthesize IGF gene expression and dietary Mn help in this process, the Mn diet is responsible for growth enhancement and biomolecular function in the cell of aquatic organism. The stressors (As + NH_3_ + T, NH_3_ + T, As + NH_3_, NH_3_, and As) drastically inhibited the growth performance (final weight gain %, FCR, SGR, PER, DGI, TGC, and RFI) of the fish might be due to arsenic and ammonia toxicity, and high-temperature stress reduces the feed intake and metabolic rate, which was reported by our previous study^2^. Interestingly, the Mn diet improved growth performance could be due to the role of Mn in improving feed efficiency, feed utilization, growth rate, and immunity of the fish. It also improved the specific growth rate, daily growth index %, relative feed intake and protein efficiency in the fish^90^. Moreover, the deficiency and inadequate supply of Mn result in reduced growth rate, reduced feed intake, skeletal abnormalities such as dwarfism and cataract in fish^91^. Mn has also provided the uptake of glucose, insulin receptors and triglyceride synthesis^92^. However, the dietary Mn at optimum levels is beneficial for growth enhancement of fish reread under control and stressed environment.

The present study revealed that ammonia and arsenic toxicity and high-temperature stress elevated the LPO level in the liver and kidney tissues might be due to the formation of ROS by stressors. A free radical producing system mainly generates it. Excessive ROS generation may cause oxidative stress and damage critical biomolecules, resulting in deleterious biological effects^93^. Moreover, the dietary Mn at 4 and 8 mg kg^−1^ diet remarkably reduced the ROS and LPO levels. Similarly, the muscle and brain Vit C were noticeably elevated by dietary Mn at 4 and 8 mg kg^−1^ diet. It is crucial for collagen synthesis and in metabolism of biomolecules, including steroids and detoxification of xenobiotics^94^. Therefore, Mn is important in maintaining the Vit C in fish tissues. The blood profiling viz. Hb, WBC and RBC were important component which were altered by arsenic, ammonia and high temperature, whereas, the dietary Mn at 8 mg kg^−1^ diet was corrected the count of Hb, WBC and RBC. Hb helps in aerobic metabolism, distribution of the gases and maintenance of the physiological attributes in the fish via fish growth and health^95^. The RBC helped absorb oxygen through gill and circulated in the different tissues in the body. Stress changed not only the RBC count but also the shape of the RBC. Further, the WBC is an important component for acquired and innate immune response. It constituted eosinophils, neutrophils, lymphocytes, monocytes, and basophils. Hence, the dietary Mn enhances the WBC count in fish reared in control or stress conditions.

NBT, blood glucose, total protein, albumin, globulin, A:G ratio, and MPO are important attributes of immunity. In the present study, stressors altered the immunity, whereas the dietary Mn approved the immunity in the fish. NBT indicates the health of the fish as elevated levels mention higher immunity. It involved the phagocytes for intercellular superoxide radicals produced by leucocytes^96^. Moreover, the globulin are also major component and four types such as α_1_, α_2_, β and γ^97^, which the gamma globulin is essential for blood immunological protein^98^. Further, albumin helps in transportation of hormones, metal, bilirubin, drug and vitamin. It also regulates the free available hormones^99^ and fat metabolism. Interestingly, the supplementation of Mn diet enhances the production of B-lymphocytes and it maintained the higher immunity of the fish. However, MPO is the haemoprotein and important during respiratory burst using H_2_O_2_ to produce hypochlorous acid^100^. Hypochlorous acid is a potent oxidant that elicit the cytotoxic effect on bacterial cells^101^. Moreover, the Mn containing diets helps in released of neutrophils and O_2_ derived species (H_2_O_2_) and H_2_O_2_ to oxidize Cl^-^ ions to form HOCl. Moreover, the blood glucose are indicators for good health and Mn diet improved the BG level after exposure to stressors. The role of Mn in regulating blood glucose might be due to, it enhances the gluconeogenesis viz. synthesis of glucose from non-carbohydrate source mainly protein and amino acid, and the enhancement of secretion of catecholamine^102^.

The carbohydrate and protein metabolic enzymes viz. LDH, MDH, ALT and AST activities were notably elevated whereas the dietary Mn at 4 and 8 mg kg^−1^ diet reduced the activities. This might be due to Mn fulfilled the energy demand during stress conditions, and hence, it reduces the activities of LDH and MDH. Notably, the LDH is the glycolytic enzyme that catalyzes the interconversion of pyruvate and lactate using the nicotinamide adenine dinucleotide (NAD) as a coenzyme. Moreover, the MDH is the limiting enzyme for the oxidative catabolism of carbohydrates^103^. The ALT and AST activities were also reduced by the Mn diet, possibly because Mn is required for many cofactors for biomolecular enzymes.

The stressors (As + NH_3_ + T, As + T, As + NH_3_, NH_3_, and As) significantly inhibited AChE activities might be due to arsenic, ammonia, and high temperature preventing the hydrolysis of acetylcholine^58^. Moreover, acetylcholine helps dominate cholinergic synapses and neuromuscular junctions in the fish's central nervous system (CNS). This results in the hydrolysis of acetylcholine and choline after the activation of acetylcholine receptors at the postsynaptic membrane^104^. Surprisingly, the AChE activities were improved by dietary Mn. It also showed that a higher Mn diet at 12 mg kg^−1^ diet significantly inhibited AChE activities, which might be due to its nature to induced the toxicity to the postsynaptic membrane.

The stressors group, such as arsenic, ammonia toxicity, and high-temperature stress, enhances arsenic bioaccumulation in the fish, whereas the Mn diet at 8 mg kg^−1^ diet reduced the arsenic bioaccumulation. This might be due to the ability of Mn to enhance the detoxification of arsenic in all tissues. Moreover, the kidney and liver tissues had higher arsenic bioaccumulation reported in the present investigation. These results revealed that Mn could detoxify arsenic efficiently in all the tissues.

The present study also showed that dietary Mn at 8 mg kg^−1^ diet enhanced fish survival after an infection of a bacterial pathogen. The results of the present study showed the Mn diet improved the antioxidant and immunity of the fish. However, this might be the reason for the higher survival of the fish against pathogenic infection after the dietary application of Mn. It is also reported that Mn helps generate neutrophils, which enhances the effector cells function for defencing the fish against pathogenic bacteria^105^.

## Conclusion

The present study is the first report on the role of manganese (Mn) on gene regulations and biochemical regulators in response to arsenic and ammonia toxicity and high-temperature stress in *P. hypophthalmus*. The immunity, anti-oxidative status, growth performance, genotoxicity, and other stress-responsive genes were controlled and regulated by dietary Mn at 8 mg kg^−1^ diet. Mn at 8 mg kg^−1^ diet efficiently regulates cortisol, HSP 70, and apoptosis and protects against genotoxicity. Mn at 8 mg kg^−1^ diet is also efficient in enhancing the detoxification of arsenic in different fish tissues. Moreover, the results revealed that Mn at 8 mg kg^−1^ efficiently controls the gene regulation involved in the multiple stressors (As + NH_3_ + T). Indeed, dietary Mn at 8 mg kg^−1^ diet improved gene regulation, maintained fish hemostasis, and noticeably reduced the bioaccumulation of arsenic in fish tissues. Overall results of the present investigation concluded that Mn at 8 mg kg^−1^ diet should be included in the fish diet to maintain gene regulation of the NFkB signaling pathway and mitigate the multiple stresses in fish.

## Data Availability

The datasets generated during and/or analysed during the current study are available from the corresponding author on reasonable request.
